# A Systematic Review and Quality Assessment of Pharmacoeconomic Publications for China Compared to Internationally: Is the Quality of Evidence-Base Sufficient for Health Technology Assessment?

**DOI:** 10.34172/ijhpm.8656

**Published:** 2025-04-28

**Authors:** Zhixin Fan, Xu Si, Zhongxiang Wang, Liwei Zhang, Junyang Liu, Qing He, Matthew Franklin, Qiang Sun, Jia Yin

**Affiliations:** ^1^Department of Social Medicine and Health Management, School of Public Health, Cheeloo College of Medicine, Shandong University, Jinan, China.; ^2^NHC Key Lab of Health Economics and Policy Research, Shandong University, Jinan, China.; ^3^Center for Health Management and Policy Research, Shandong University, Shandong Provincial Key New Think Tank, Jinan, China.; ^4^Zhucheng Shiqiaozi Health Hospital, Zhucheng, China.; ^5^Health Economics and Decision Science (HEDS), School of Health and Related Research (ScHARR), University of Sheffield, Sheffield, UK.; ^6^China National Health Development Research Center, Beijing, China.

**Keywords:** Quality Assessment, HTA, Pharmacoecomics, Systematic Review, Umbrella Review

## Abstract

**Background:**

Pharmacoeconomic evaluations are becoming more important in China, and their research quality directly impacts government decisions, deserving extra attention. To summarize the quality of pharmacoeconomic publications for China compared to internationally and to identify areas for improvement both from a China-specific and international perspective.

**Methods:**

First, we conducted a systematic review of pharmacoeconomic publications for China, with subsequent reporting quality assessment based on the Consolidated Health Economic Evaluation Reporting Standards (CHEERS) checklist. Second, we conducted an umbrella review of pharmacoeconomic publications internationally which used a similar quality assessment. We extracted the CHEERS checklist scores for each study and converted them to percentages to facilitate comparison of results.

**Results:**

CHEERS 2022 instrument was used to evaluate the quality of 154 pharmacoeconomic publications by Chinese scholars. Across these articles, the average quality score was 61.0%, indicating a moderate level of quality on average. There were 27 (17.5%) high-quality articles, 85 moderate quality articles (55.2%) and 42 low-quality (27.3%) articles. Out of 28 scoring items, those included in the methods section such as: health economic analysis plan, characterizing heterogeneity, characterizing distributional effects, approach to engagement with patients and others affected by the study, got low scores. In addition to the generally lower scores of international articles on items 9 (Time horizon), 18 (Characterizing heterogeneity) and 24 (Effect of uncertainty), Chinese articles also scored lower than international articles on items included in the methods and other relevant information section, eg, health economic analysis plan, perspective, discount rate, analytics and assumptions, characterizing distributional effects, approach to engagement with patients and others affected by the study, source of funding, and conflicts of interest.

**Conclusion:**

The quality of China’s pharmacoeconomic publications has been improving year by year since the establishment of the National Healthcare Security Administration (NHSA) in 2018, but there is still a quality gap with similar international publications which requires further focus and improvement in study conduct and reporting standards for the evidence-base to be sufficient for health technology assessment (HTA).

## Background

 Medical expenditures are rising rapidly, partly attributed to the upgrading of new and patented drugs, alongside increasing and diversifying healthcare needs associated with an ageing population.^1,2^ Faced with limited health resources, maximizing health benefits through efficient resource allocation has always been a challenge for policy-makers worldwide. As such, health technology assessment (HTA) has become a crucial decision-making tool to provide an evidence-base on which governments and governing agencies (eg, reimbursement agencies) can inform their decisions about how to allocate finite healthcare resources appropriately and efficiently. It is a multidisciplinary process that uses explicit methods to determine the value of a health technology, including medicine, vaccine, procedure, etc, at different points in its lifecycle.^3^ HTA helps policy-makers in understanding the cost-effectiveness of various health technologies, facilitating the phase-out of outdated ones, supporting innovative developments, and ultimately optimizing resource allocation and utilization, while driving continuous improvement and optimization of medical services.

 In the fields where HTA is applied, drug prices and reimbursement are of greater concern to the governments around the world. The Australian government was the first to announce that pharmacoeconomic evaluation evidence would be required in submissions to the Pharmaceutical Benefits Advisory Committee, for which a mandatory evaluation guideline was produced. After evaluation, Pharmaceutical Benefits Advisory Committee submits a recommended price range for new drugs to be included in the Pharmaceutical Benefits Scheme to the Ministry for Health and Aging, and then price negotiations commence.^4^ In the United Kingdom, National Institute for Health and Care Excellence conducts pharmacoeconomic evaluations to determine cost-effective drug pricing. Based on this pricing, the National Health Service negotiates with pharmaceutical companies to set the prices of drugs within the National Health Service reimbursement list.^5,6^ After, more and more countries in the European Union, New Zealand, Canada, the United States, Latin America, and Asia have also taken an interest in pharmacoeconomic evaluation.^4,7^

 The concept of pharmacoeconomics was first introduced to China in the 1990s, but was not widely applied until the establishment of the National Healthcare Security Administration (NHSA) in 2018.^8^ The NHSA explicitly adopted pharmacoeconomic evaluation evidence as one of the supporting evidences required for drug reimbursement negotiations and national healthcare insurance catalogues adjustment, similar to other HTA-related bodies internationally.^9^ However, pharmacoeconomic evaluation is still nascent in China. To identify areas for improvement both from a China-specific and international perspective, we conducted a systematic review to summarize the quality of pharmacoeconomic publications for China since 2018, and then conducted an umbrella review which used a similar quality assessment to compared the quality of pharmacoeconomic publications for China to internationally.

## Methods

 The systematic review was conducted according to the guidelines of the Preferred Reporting Items for Systematic Reviews and Meta-Analyses (PRISMA). An umbrella review is a review of reviews, and its most typical characteristic is that this type of evidence synthesis only considers the inclusion of the highest level of evidence, namely systematic reviews.^10^ Conducting an umbrella review can provide a rapid method for examining evidence, allowing for the comparison and contrast of the results of individual reviews. This approach addresses a broad and high-quality evidence base related to a particular topic, thereby providing healthcare decision-makers with the evidence they need. It is increasingly being applied widely. In this study, conducting an umbrella review allows for good international comparisons and the identification of areas for improvement.

###  Search Strategy

 Articles published between 2018 and 2023 were systematically searched. For the systematic review, we searched databases of CNKI, Wanfang, VIP, PubMed, and Web of Science databases to retrieve pharmacoeconomic evaluations published by Chinese scholars. The following keywords were used in the search terms: “pharmacoeconomic,” “pharmacoeconomic evaluation,” “economics,” “economic evaluation,” “health economics,” “cost-effectiveness,” “cost-benefit,” “cost-utility,” “systematic review,” “review,” ‘China,” and “Chinese.”

 For the umbrella review, keywords such as “pharmacoeconomic,” “pharmacoeconomic evaluation,” “health economics,” “cost-effectiveness,” “cost-benefit,” “cost-utility,” “systematic review,” “review,” “methodology,” “methodological quality,” “quality,” “quality evaluation,” and “quality assessment” were used to search for articles in databases of PubMed and Web of Science databases. See Supplementary file 1 for more details.

###  Selection of Studies

 In the systematic review section, the inclusion criteria were: (1) study language was limited in Chinese and English; (2) compares drugs or pharmaceuticals; (3) original study on pharmacoeconomics; and (4) conducted in China by Chinese scholars; the exclusion criteria were: (1) conference papers, dissertation, conference abstracts, or other non-peer-reviewed publications; (2) budget impact analysis; (3) theoretical studies and reviews in pharmacoeconomics; (4) full text not available; (5) articles in Chinese not published in core journals or national journals; (6) studies by Chinese scholars that are not conducted in China. Among Chinese journals, core journals or national journals are professional journals of high quality that represent the level of development of the professional discipline and are valued by readers of the discipline. The articles in these journals are rigorously peer-reviewed to ensure that the included Chinese articles were comparable to international articles. In this study, we restrict the source of Chinese journals according to the Catalogue of National Chinese Core Journals (2023 Edition) and official website of the journals in 2024 to identify problems in a more targeted manner.

 In the umbrella review section, the inclusion criteria were: (1) study language was limited in Chinese and English; (2) compares drugs or pharmaceuticals; (3) original study on pharmacoeconomics; the exclusion criteria were: (1) conference papers, dissertation, conference abstracts, or other non-peer-reviewed publications; (2) budget impact analysis; (3) theoretical studies and reviews in pharmacoeconomics; (4) full text not available; (5) reviews used any form of quality assessment except Consolidated Health Economic Evaluation Reporting Standards (CHEERS) statement.

 The selection process was divided into two steps: primary screening and re-screening, first by the title and abstract of each article and then by the full text. Each step was performed by two researchers (ZF, XS, JL, and LZ). Any disagreements at this stage were resolved through consultation with a third party (QS).

###  Quality Assessment and Associated Scoring System

 CHEERS statement focuses on what has been reported for the economic evaluation, which is a useful and practical tool to improve reporting and, in turn, health and healthcare decisions. In this systematic review, we used the 28-item CHEERS 2022 statement to evaluate the reporting standards of the included articles.^11^ CHEER 2022 includes 28 items, reflecting the quality of title (item 1), abstract (item 2), introduction (item 3), methods (item 4-21), results (item 22-25), discussion (item 26), other relevant information (item 27-28). For the purpose of using a numerical scoring system to suggest the quality of the reporting standards of the include article, we used a scoring methods suggested by Yu et al; that is, we allocated a score of 1 for “yes,” 0.5 for “partially,” and 0 for “no”/“not applicable” to each of the CHEERs items.^12,13^ Furthermore, a score of 1 was allocated to items that the study explicitly stated “not applicable” and provided a reason, whereas a score of 0.5 was allocated for studies that mentioned “not applicable” without any explanation. If the item was not mentioned in the study, a score of 0 was allocated. Subsequently, the allocated score was divided by the total score from 0 to 28, such that the allocated scores were converted into percentages to reflect the quality of the studies. To further aid with describing the quality of the studies, studies with a score of 75% or more were regarded as high-quality, 50%-74% were moderate quality, and those below 50% were considered low-quality.^11,14^ See Supplementary files 2 and 3 for more details.

 The quality assessment was performed by two researchers independently. Any disagreement was resolved through discussion. If a consensus could not be reached, a third researcher was consulted. In addition, we extracted the CHEERS checklist scores for each studies included the umbrella review and converted them to percentages to facilitate comparison of results. We also used the Measurement Tool to Assess systematic Reviews (AMSTAR) 2 to evaluate the methodological quality of studies included in the umbrella review, as it is more specific and sensitive in identifying the methodological quality of systematic reviews.^15^

## Results

###  Systematic Review of Pharmacoeconomic Evaluations Conducted in China

####  Basic Characteristics of Included Articles

**Figure 1:**
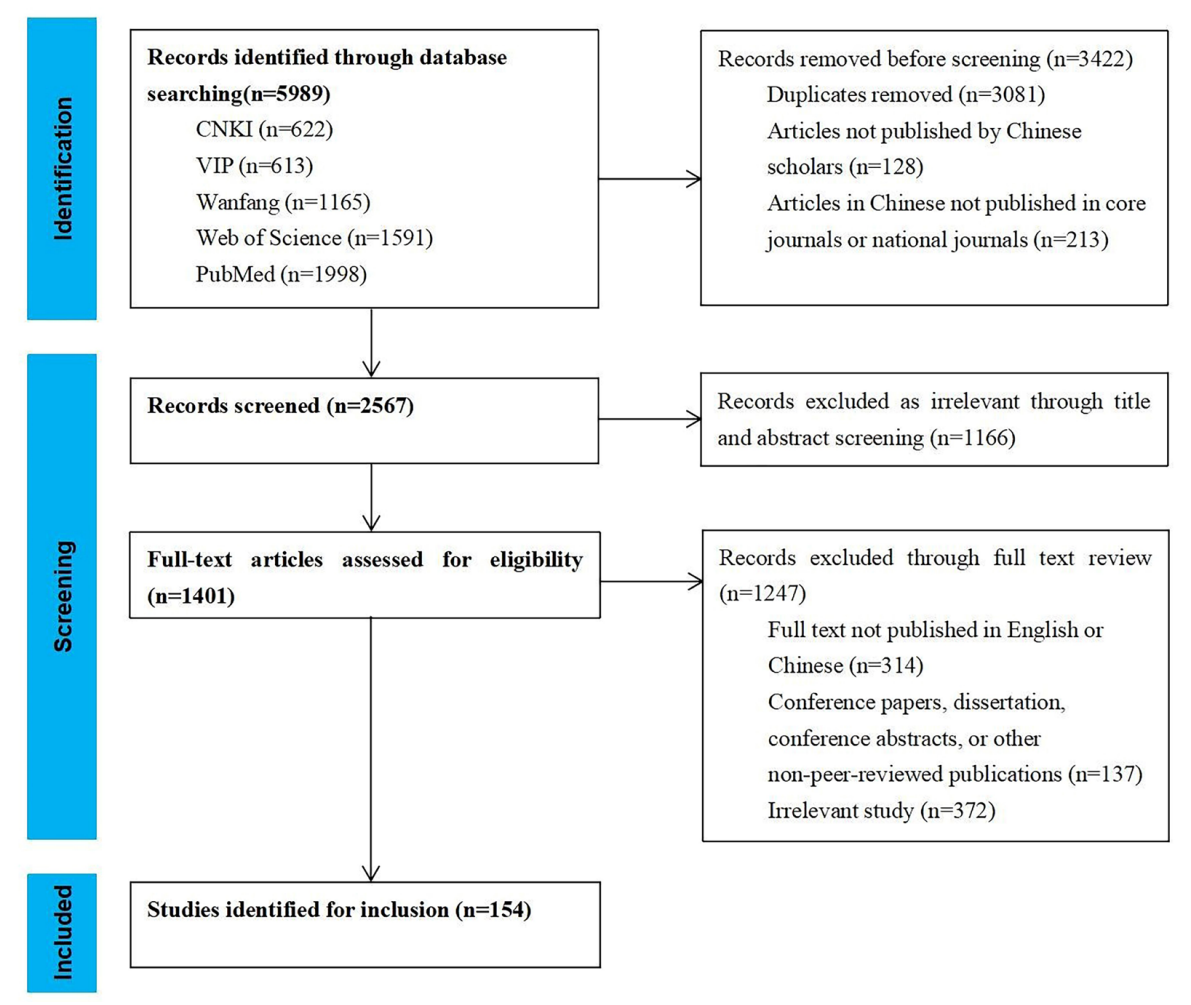
PRISMA 2020 Flow Diagram for Included Studies in Systematic Reviews. Abbreviation: PRISMA, Preferred Reporting Items for Systematic Reviews and Meta-Analyses.

 A total of 154 articles published by Chinese scholars were included based on our inclusion and exclusion criteria (Figure 1), including 90 Chinese articles and 64 non-Chinese articles. We have looked at the first author affiliation of articles and the authors only from medical institutions had the highest proportion, accounting for 49.4%, followed by universities (31.8%). The included articles covered several types of diseases, with most focused on tumors (39.6%) followed by cardiovascular diseases (15.6%).

 Of the 154 included studies, the most used evaluation perspective was a healthcare system perspective (34.4%) with the least used a societal perspective (5.1%); 31.8% of studies did not specify the evaluation perspective. Most articles (38.3%) used a discount rate of 5%, which is the recommended rate for China. 53 articles (34.4%) did not report a discount rate, of which 14 studies did not need to consider discounting due to the time horizon of the study being less than 1 year, and the remaining 39 articles did not reasonably consider discounting. Sensitivity analysis methods mainly include one-way sensitivity analysis (25.3%), analysis using both methods such as multi-factor sensitivity analysis, probabilistic sensitivity analysis (59.7%) and other sensitivity analyses (1.9%). The vast majority of the studies (65.6%) used cost-effectiveness analysis as the research method, followed by studies using cost-utility analysis or both methods. Out of 154 articles, more studies used Markov (29.2%), partitioned survival model (14.9%) and decision tree (13.6%) as evaluation models, while 55 (35.7%) articles did not use any modelling-based economic evaluation.

####  Quality Assessment

**Figure 2:**
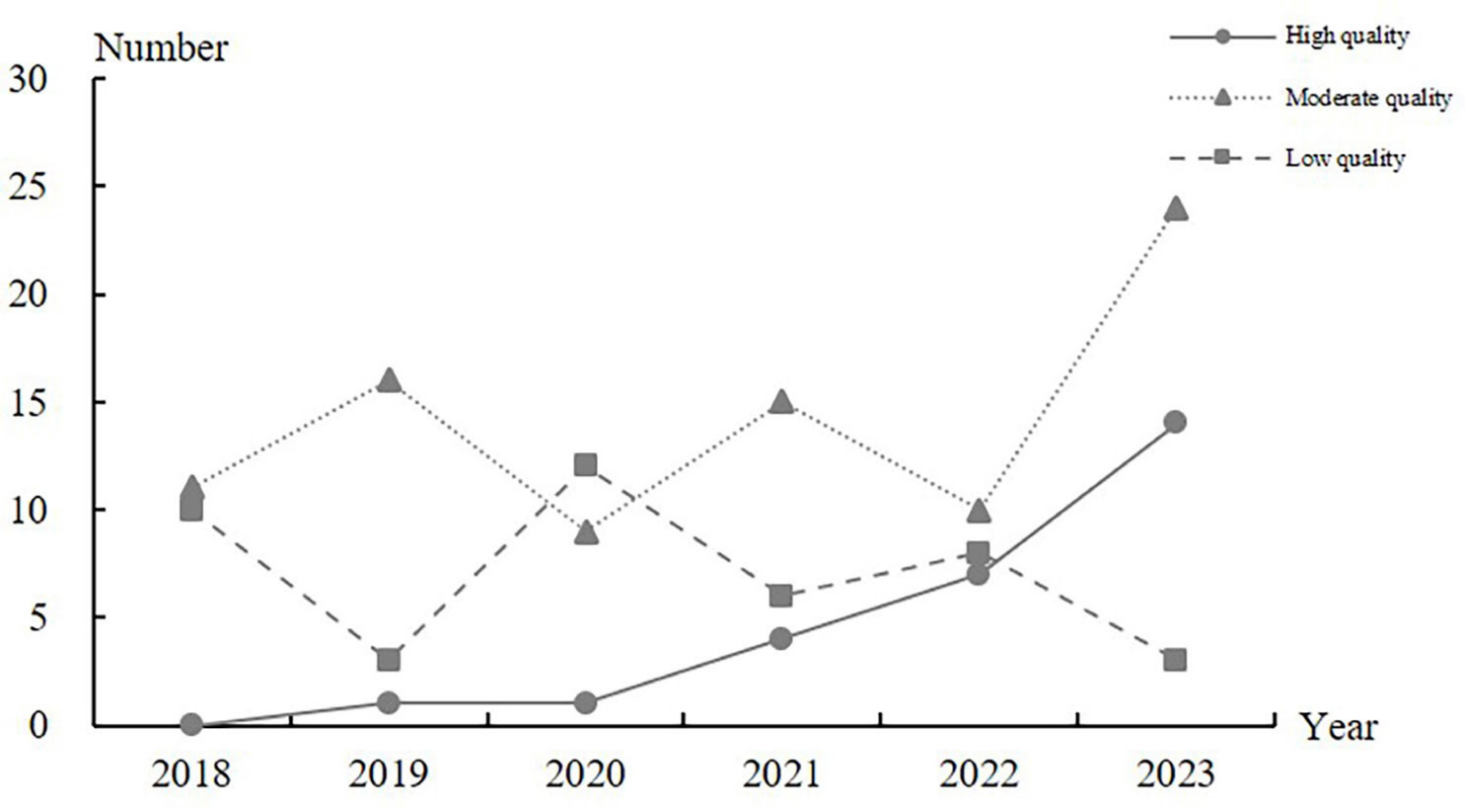
Quality of Pharmacoeconomic Evaluation in China From 2018 to 2023.

 By applying a numerical scoring system to the CHEERS 2022 statement to aid with describing the quality of the included studies, the average quality score of the 154 economic evaluation studies by Chinese scholars was 61.0% (ie, on average, these studies were of moderate quality). There were 27 (17.5%) high-quality articles and 42 low-quality (27.3%) articles. The number of high-quality articles showed an increasing trend year-by-year, from 0% published in 2018 to 34.2% published in 2023 (Figure 2).

**Figure 3:**
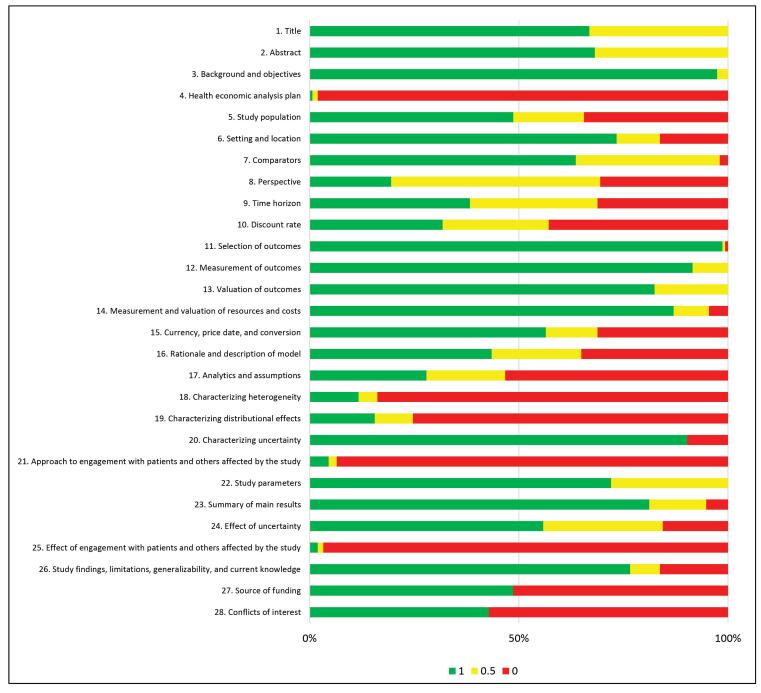
Average Score for CHEERS 2022 Items. Abbreviation: CHEERS, Consolidated Health Economic Evaluation Reporting Standards.

 None of the 154 studies met all of the CHEERS reporting standard criteria. Out of 28 scoring items, those included in the methods section such as: “health economic analysis plan,” “characterizing heterogeneity,” “characterizing distributional effects,” “approach to engagement with patients and others affected by the study,” received low scores while items 3 (Background and objectives) and 11-14 (“Selection of outcomes”; “measurement of outcomes”; “valuation of outcomes”; “measurement and valuation of resources and costs”) achieved high scores (Figure 3).

**Figure 4:**
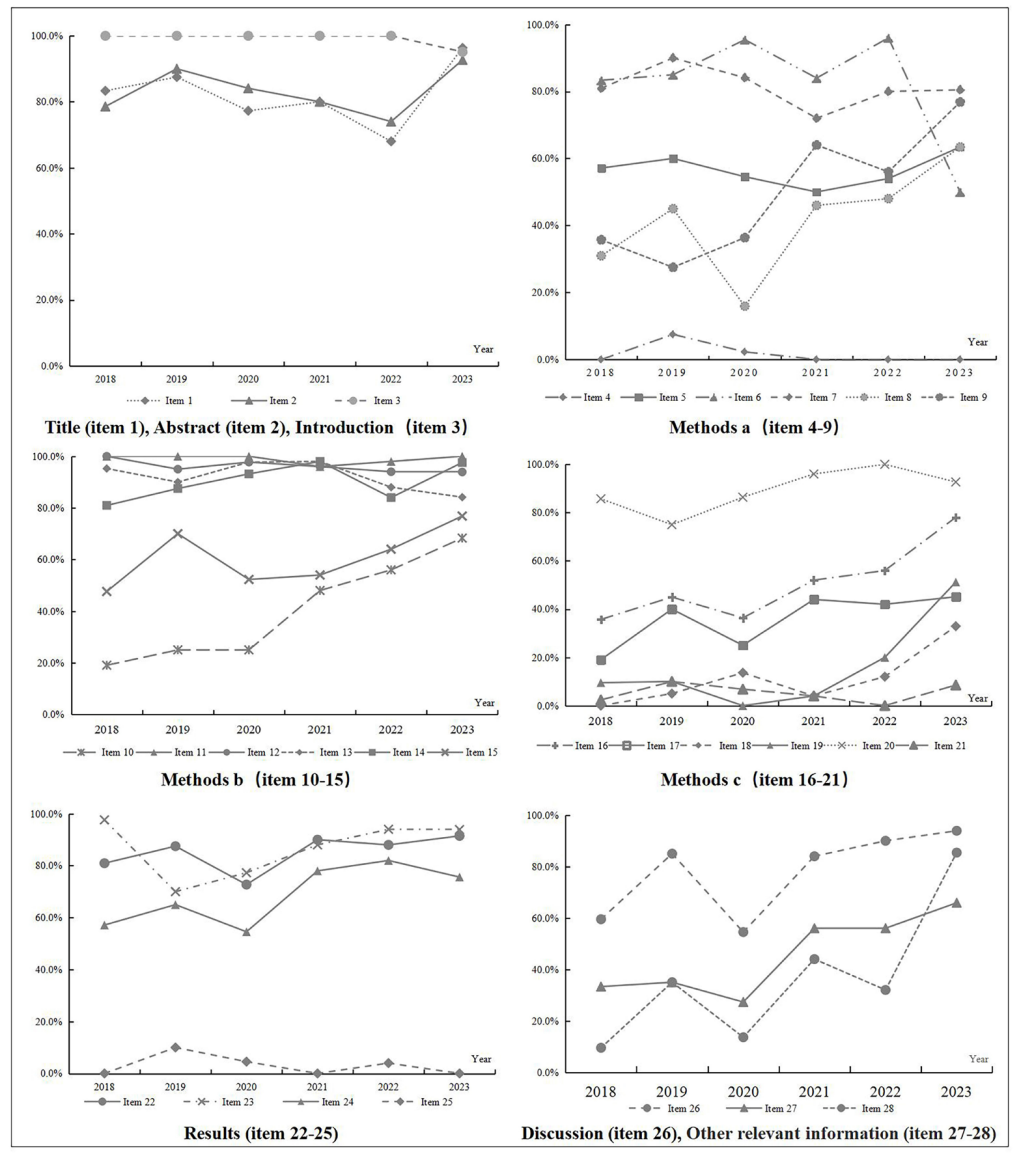
Specific Score for CHEERS 2022 Items in 2018-2023. Abbreviation: CHEERS, Consolidated Health Economic Evaluation Reporting Standards.

 We analyzed changes in the quality scores of each item. The scores for those included in the abstract and introduction section showed a decreasing trend after 2019 and an increase after 2022. In the methods section, item 4 (Health economic analysis plan) had the lowest score and did not fluctuate significantly from 2018 to 2023. There was a marked and fluctuating increase in the scores for items 8-10 (Perspective; time horizon; discount rate) and 15-19 (“Currency, price date, and conversion”; “rationale and description of model”; “analytics and assumptions”; “characterizing heterogeneity”; and “characterizing distributional effects”). In the results section, the scores for item 25 (“Effect of engagement with patients and others affected by the study”) were consistently low from 2018-2023, fluctuating between 0% and 10%, while item 22 (Study parameters) and 24 (Effect of uncertainty) tended to fluctuate and increase from year to year. In the discussion and other relevant information section, the scores for those showed fluctuating growth with significant increases (Figure 4).

###  Umbrella Review of Pharmacoeconomic Evaluation Reviews Internationally

**Figure 5:**
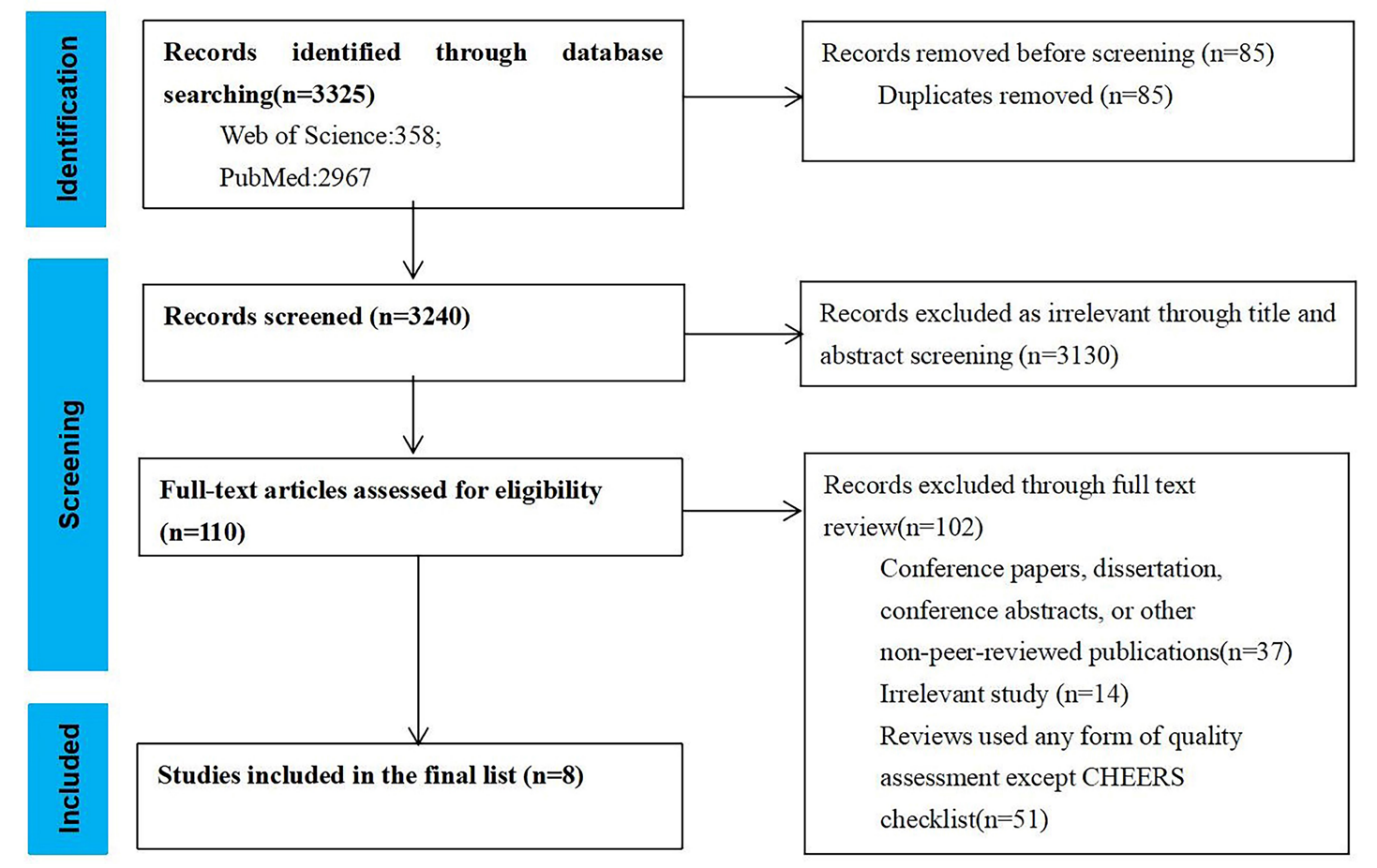
PRISMA 2020 Flow Diagram for Included Studies in Umbrella Reviews. Abbreviations: PRISMA, Preferred Reporting Items for Systematic Reviews and Meta-Analyses; CHEERS, Consolidated Health Economic Evaluation Reporting Standards.

 A total of 3325 relevant reviews were retrieved from Web of Science and PubMed, and 85 duplicates were deleted. 3130 articles were excluded by two researchers after reading the titles, abstracts, etc of the reviews, and first deleted irrelevant articles according to the inclusion and exclusion criteria, and 110 articles remained. Then 102 articles were excluded after careful reading of the full text of the reviews, and finally eight articles were included (Figure 5), including six articles using CHEERS (2013) and two articles using CHEERS (2022). We evaluated the quality of eight articles using AMSTAR 2 instrument, and the results showed that the quality of the articles was relatively high. Further details are provided in Supplementary file 4.

 In eight reviews, the most common diseases studied were cancer, respiratory, and cardiovascular diseases. The maximum number of articles included could be up to 21 and the minimum was 6, and the average number of articles included in each review was 13. Geographically, the eight reviews included studies from North America, Europe, and Asia, with a particular focus on the United States, United Kingdom, Canada, and Japan. In the pharmacoeconomic publications covered by these 8 reviews, the healthcare system and payer perspectives were most common, societal rarely adopted; over 2/3 of studies included direct medical costs, with few covering indirect costs; Markov and decision tree models were most frequently used. Most studies used one-way or the probabilistic sensitivity analysis. See Supplementary file 5 for more details.

**Figure 6:**
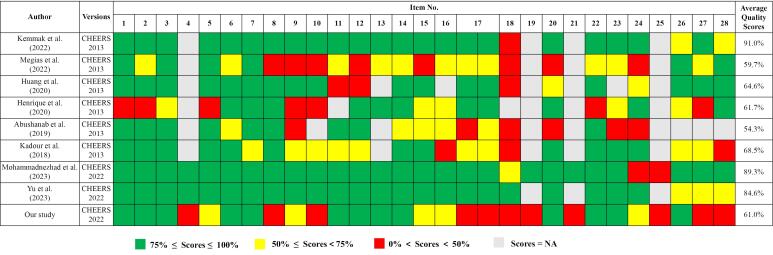
Comparison of the Quality of Pharmacoeconomic Studies in China and Internationally. Note: The difference between CHEERS (2013) and CHEERS (2022) is mainly the addition of items 4, 19, 21, and 25. The meanings of the rest of the entries remained unchanged, and only the order of the entries was changed, so that the entries in Figure 5 were based on the entries of CHEERS (2022) as the default. Abbreviations: CHEERS, Consolidated Health Economic Evaluation Reporting Standards; NA, not available.

 The highest score was 91.0% and the lowest score was 54.3%. Compared with other countries, the quality of pharmacoeconomic evaluation studies in China was relatively low. Internationally, most studies generally scored low on items 9 (Time horizon), 18 (Characterizing heterogeneity) and 24 (Effect of uncertainty), the same problem was identified for our included Chinese studies. Also, Chinese studies scored lower on item 4 (Health economic analysis plan), 8 (Perspective), 10 (Discount rate), 17 (Analytics and assumptions), 19 (Characterizing distributional effects), 21 (Approach to engagement with patients and others affected by the study), 27 (Source of funding) and 28 (Conflicts of interest) compared to international studies (Figure 6).

## Discussion

 Our findings suggest that the quality of pharmacoeconomic publications for China has improved at a reasonable rate since the establishment of NHSA in 2018; however, there are still some gaps in the quality of Chinese studies compared to relevant international studies as identified by umbrella review as a pragmatic option for making this comparison.

 Our study indicates that many published studies still do not clearly report an evaluation perspective and discount rates. Many articles did not state an evaluation perspective, or, when they did, misreported the evaluation perspective; for example, some articles stated the evaluation perspective as a “societal perspective,” but only include direct medical costs or direct costs, without considering indirect costs (62.5%).^16^ Similar conclusions were drawn by Desai and colleagues in their evaluation of the quality of pharmacoeconomic research in India.^17^ In addition, a discount rate was not considered for some studies with a study time horizon of more than one year. Some researchers did not include time in measuring cost and health outcomes.

 Additionally, the ambiguity of the assumptions of the evaluation model is also of concern. Many studies were limited to short-term follow-up (less than one year). In order to determine the long-term cost-effectiveness of drugs, modelling-based economic evaluation have become valuable instruments. Choosing a variety of prediction models can facilitate accurate extrapolation and short-term prediction. However, the application of mathematical decision-analytic models is typically dependent on many assumptions, and vague assumptions may somewhat compromise the validity of any subsequent results.

 Our study also found that the quality of studies using the modelling-based economic evaluation was much higher than those not using it, which is similar to the results of other studies.^18^ More than one third of the studies published by Chinese researchers did not use modelling-based economic evaluation. Cost-utility analysis and studies using the partitioned survival model had the highest proportion of high quality, which were mostly published by university researchers; researchers from medical institution tended to favour cost-effectiveness analysis and did not use modelling-based economic evaluation. This reflects to some extent that their knowledge of pharmacoeconomic theory and methodology is inadequate, including biased understanding and misuse. Previous studies have reached similar conclusions.^19^ The methodology of pharmacoeconomic evaluation need to be further developed in China.

 Representing heterogeneity and distributional effects have recently received extensive attention from scholars. Our study suggests that the current global pharmacoeconomic research has under considered both characterization of heterogeneity and distributional effects. A study in the United States showed that the cost-effectiveness of statins varied widely in populations with different levels of vascular risk.^20^ Consideration should be given to how heterogeneity in study outcomes arises in order to appropriately explore and report on the impact of different types of heterogeneity. Distributional effects incorporate health inequities into the analytical framework of economic evaluations of health-related interventions to compensate for the inability of traditional cost-effectiveness analyses to answer the question, “Does the intervention improve/worsen health inequities?” Researchers can provide details of the distribution of who benefits most and who bears the greatest burden (opportunity cost) based on equity-related social variables (eg, socioeconomic status, ethnicity, and region) and disease type variables (eg, disease severity, rarity, and disability).^21^ And that means that the importance of data availability cannot be overstated. However, now it is a major challenge not only for China, but also common to most developing countries. In the absence of localized data such as health utility values based on domestic population measurements and transition probabilities for different disease states, the citation of literature from other regions results in poor data representativeness and inadequate extrapolation of results, which in turn affects the scientificity and rationality of decision-making. We call on Chinese researchers to conduct more re-evaluation studies based on real-world data and to collect localized baseline data to provide more realistic and reliable information for future evaluation studies, which are also worthy of consideration by other developing countries.

 Overall, pharmacoeconomic publications for China has performed poorly in terms of financial disclosure and declaration of interest compared to international studies. Chinese articles performed worse than non-Chinese articles. This may be because many non-Chinese journals require researchers to provide a statement of relevant funding disclosure when accepting articles, thereby increasing the transparency of research. However, Chinese journals mostly overlook this issue. The use/reference to health economics analysis plans and the focus on stakeholder engagement are new to the CHEERS 2022 checklist, with a strong emphasis on transparency in the research process, the evaluation of research methods, and outcomes for different stakeholders especially patients and general public. This places greater demands on future pharmacoeconomic evaluation studies and is something that researchers will need to think about more in the future.

 The development and quality of pharmacoeconomic evaluation have been driven in part by the need for government decision-making and the introduction of the latest guidelines. Currently, the NHSA explicitly adopts pharmacoeconomic evaluation reports as one of the supporting evidences in China. Policy adjustments have increased the need for pharmacoeconomic evaluations among healthcare providers, policy-makers, and pharmaceutical companies in areas such as drug procurement, adjustment of national healthcare insurance catalogues, post-marketing drug evaluation, new drug application access, and drug pricing. Many studies show that the quality of pharmacoeconomic evaluations is higher in developed countries than in most developing countries, which can also be confirmed to a certain extent in the development history of pharmacoeconomics in different countries.^22-24^ An increasing number of countries have incorporated pharmacoeconomic evaluation into government decision-making and are constantly revising and updating guidelines.^5,6,25^ For China, the current guideline is the China Pharmacoeconomic Evaluation Guideline 2020,^26^ which provides a relative clear research framework and design criteria for pharmacoeconomic research in China. In this study, we found that low scores for some items do not indicate missing; but they often result from unclear reporting, such as the lack of explanation for choosing research perspectives, discount rate, methodological models et al. Partly due to researchers’ misunderstanding of methodology, and partly due to their unawareness of the need to state these contents. We need to acknowledge the absence of more specific descriptions and guidance concerning certain aspects within the guideline. For instance, it recommends prioritizing social and health systems, yet offer no explicit definitions. The Dutch guidelines describe in detail the costs that should be included from a societal perspective and require the inclusion of patient and family costs, such as travel costs and unpaid work.^27^ Danish, Canadian and Indonesian guidelines also list and provide detailed descriptions of all costs to be included from provider, patient, payer, and societal perspectives.^28-30^ Similar problems also include model assumptions, heterogeneity analysis, etc, are challenging for researchers, as the guidelines lack detailed references, necessitating further improvement in the future. Furthermore, relevant researchers should correctly understand the contents of guidelines, and scientifically design research protocols, especially focusing on the methodology section. We also encourage cross-regional and cross-institutional collaborations, and attempt to establish independent third-party evaluation institutions and develop criteria for assessing the quality of pharmacoeconomic evaluations objectively and scientifically, thereby compensating for individual researchers’ limitations and providing high-quality decision-making support for government policy-makers.

 There are some limitations to this study. First, despite searching for published articles using multiple search engines, some articles may have been inadvertently excluded. Government reports, or unpublished articles were not included in the evaluation, so there was a degree of publication bias in this study. Using CHEERS 2022 to evaluate the quality of pharmacoeconomic evaluation based on a numerical scoring system and somewhat arbitrary grading system was useful for descriptive purposes, but is not an established way to use CHEERS for grading the reporting quality of studies; although, its use and usefulness is evidence in this study and previous studies.^11^ Additionally, CHEERS focuses on reporting quality standards not necessarily the actual quality of the conducted studies; however, as better reporting is potentially linked with knowledge and ability to conduct the study appropriately, we have assumed an association between reporting standards and the conduct quality of the related study. Finally, grading the quality of the identified studies has some subjectively such that other reviewers may have scored the articles differently, resulting in slight differences in the final evaluation results; although, for this study the two reviewers fully communicated and agreed on the content of each entry beforehand, and agreed on any disagreements through third-party negotiation to ensure good scoring reliability.

## Conclusions

 Since the establishment of NHSA in 2018, the reporting quality of pharmacoeconomics in China has shown improvement year-on-year. There is still a gap between the quality of the pharmacoeconomic publications for China compared to our identified international studies. In particular, studies need to be better at considering, using and reporting relevant research perspectives, discount rates, model analysis assumptions, distributional effects, and research transparency. Those conducting and reporting on pharmacoeconomic evaluations should seek to better use and report the above key issues according to guidelines (internationally and country-specific), to ensure that the reported evidence-base is sufficient for HTA in China and internationally.

## Ethical issues

 Not applicable.

## Conflicts of interest

 Authors declare that they have no conflicts of interest.

## Supplementary files


Supplementary file 1 describes the search strategies and basic information of the studies included in the systematic review.

Supplementary file 2 describes the CHEERS 2022 scores for the systematic review of the included studies.

Supplementary file 3 describes the lists of studies included in the systematic review.

Supplementary file 4 describes the CHEERS 2013/2022 checklist scores and AMSTAR 2 scores for the articles included in the umbrella review.

Supplementary file 5 describes the lists of studies included in the umbrella review and the basic information of them.

## References

[1] Kesselheim AS, Avorn J, Sarpatwari A (2016). The high cost of prescription drugs in the United States: origins and prospects for reform. JAMA.

[2] Hasegawa M, Komoto S, Shiroiwa T, Fukuda T (2020). Formal implementation of cost-effectiveness evaluations in Japan: a unique health technology assessment system. Value Health.

[3] O’Rourke B, Oortwijn W, Schuller T (2020). Announcing the new definition of health technology assessment. Value Health.

[4] Drummond M (2013). Twenty years of using economic evaluations for drug reimbursement decisions: what has been achieved?. J Health Polit Policy Law.

[5] Ikegami N, Drummond M, Fukuhara S, Nishimura S, Torrance GW, Schubert F (2002). Why has the use of health economic evaluation in Japan lagged behind that in other developed countries?. Pharmacoeconomics.

[6] Drummond M, Dubois D, Garattini L (1999). Current trends in the use of pharmacoeconomics and outcomes research in Europe. Value Health.

[7] Yang BM (2009). The future of health technology assessment in healthcare decision making in Asia. Pharmacoeconomics.

[8] Wu J, Sun L, Liu G (2008). Current situation of pharmacoeconomics in China. Chinese Pharmacoeconomics.

[9] Announcement by the National Health Insurance Administration on the Announcement of the 2019 National Health Insurance Drug List Adjustment Work Plan. National Healthcare Security Administration. http://www.nhsa.gov.cn/art/2019/4/17/art_53_1215.html. Accessed March 23, 2024.

[10] Aromataris E, Fernandez R, Godfrey C, Holly C, Khalil H, Tungpunkom P. The Joanna Briggs Institute Reviewers’ Manual 2014: Methodology for JBI Umbrella Reviews. Australia: The Joanna Briggs Institute; 2014.

[11] Husereau D, Drummond M, Augustovski F (2022). Consolidated Health Economic Evaluation Reporting Standards 2022 (CHEERS 2022) statement: updated reporting guidance for health economic evaluations. Value Health.

[12] Yu G, Tong S, Liu J (2023). A systematic review of cost-effectiveness analyses of sequential treatment for osteoporosis. Osteoporos Int.

[13] Mohammadnezhad G, Noqani H, Rostamian P, Sattarpour M, Arabloo J (2023). Lenvatinib in the treatment of unresectable hepatocellular carcinoma: a systematic review of economic evaluations. Eur J Clin Pharmacol.

[14] Chiou CF, Hay JW, Wallace JF (2003). Development and validation of a grading system for the quality of cost-effectiveness studies. Med Care.

[15] Shea BJ, Reeves BC, Wells G (2017). AMSTAR 2: a critical appraisal tool for systematic reviews that include randomised or non-randomised studies of healthcare interventions, or both. BMJ.

[16] Jing Z, Fei L, Weiling J (2018). Cost-effectiveness analysis of cervical spondylopathy treated by Zhishu Granule based on randomized clinical trial. Chinese Journal of Pharmaceutical Economics.

[17] Desai PR, Chandwani HS, Rascati KL (2012). Assessing the quality of pharmacoeconomic studies in India: a systematic review. Pharmacoeconomics.

[18] Luo Q, Zhou L, Feng H, Hu M. Systematic assessment and quality evaluation of literatures on economic evaluation of diabetes drugs in Chinese population. China Pharmacy 2022;33(10):1225-1232. [Chinese].

[19] Feng Y, Ke X, Tang W (2021). Evaluation of the quality of Chinese literature in the pharmacoeconomic evaluation of antitumor drugs. China Journal of Pharmaceutical Economics.

[20] Heart Protection Study Collaborative Group (2009). Statin cost-effectiveness in the United States for people at different vascular risk levels. Circ Cardiovasc Qual Outcomes.

[21] Husereau D, Drummond M, Augustovski F (2022). Consolidated Health Economic Evaluation Reporting Standards (CHEERS) 2022 explanation and elaboration: a report of the ISPOR CHEERS II good practices task force. Value Health.

[22] Pandey P, Pandey RD, Shah V (2018). Evaluation of quality of pharmacoeconomic studies in Asia-Pacific region and identification of influencing variables. Value Health Reg Issues.

[23] Genuino AJ, Gloria MA, Chaikledkaew U, Reungwetwattana T, Thakkinstian A (2021). Economic evaluation of adjuvant trastuzumab therapy for HER2-positive early-stage breast cancer: systematic review and quality assessment. Expert Rev Pharmacoecon Outcomes Res.

[24] Wang L, Shi F, Guan X, Xu H, Liu J, Li H (2021). A systematic review of methods and study quality of economic evaluations for the treatment of schizophrenia. Front Public Health.

[25] Liu G, Wu EQ, Ahn J (2020). The Development of Health Technology Assessment in Asia: Current Status and Future Trends. Value Health Reg Issues.

[26] Liu GG, Hu S, Wu J, et al. China Guidelines for Pharmacoeconomic Evaluations. Beijing: China Market Press; 2020.

[27] National Health Care Institute (NHCI). Guideline for Economic Evaluations in Healthcare 2016. https://tools.ispor.org/PEguidelines/source/Netherlands_Guideline_for_economic_evaluations_in_healthcare.pdf. Accessed March 23, 2024.

[28] Medicinrådet. The Danish Medicines Council Methods Guide for Assessing New Pharmaceuticals. 2021. https://medicinraadet.dk/media/wq0dxny2/the_danish_medicines_council_methods_guide_for_assessing_new_pharmaceuticals_version_1-2_adlegacy.pdf. Accessed March 23, 2024.

[29] Canadian Agency for Drugs and Technologies in Health (CADTH). Guidelines for the Economic Evaluation of Health Technologies: Canada. 2017. https://www.cda-amc.ca/sites/default/files/pdf/guidelines_for_the_economic_evaluation_of_health_technologies_canada_4th_ed.pdf. Accessed March 23, 2024.

[30] Indonesian HTA Committee Ministry of Health, Indonesia. Health Technology Assessment (HTA) Guideline. 2017. https://adphealth.org/upload/resource/FINAL_HTA_ENG_-1.pdf. Accessed March 23, 2024.

