# Supplementary material for: A Systematic Review and Quality Assessment of Pharmacoeconomic Publications for China Compared to Internationally: Is the Quality of Evidence-Base Sufficient for Health Technology Assessment?
Source: Int J Health Policy Manag. 2025 Apr 28;14:8656. doi: 10.34172/ijhpm.8656 (PMC12257205; doi:10.34172/ijhpm.8656)
Supplement: Supplementary file 1 — describes the search strategies and basic information of the studies included in the systematic review. [file ijhpm-14-8656-s001.pdf]

Article title: A Systematic Review and Quality Assessment of Pharmacoeconomic Publications for China Compared to Internationally: Is the Quality of Evidence-Base Sufficient for Health Technology Assessment?

Journal name: International Journal of Health Policy and Management

Authors' information: Zhixin Fan[1,2,3]¶, Xu Si[1,2,3]¶, Zhongxiang Wang[4], Liwei Zhang[1,2,3], Junyang Liu[1,2,3], Qing He[1,2,3], Matthew Franklin[5], Qiang Sun[1,2,3,6]*, Jia Yin[1,2,3]*

[1]Department of Social Medicine and Health Management, School of Public Health, Cheeloo College of Medicine, Shandong University, Jinan, China.

[2]NHC Key Lab of Health Economics and Policy Research, Shandong University, Jinan, China.

[3]Center for Health Management and Policy Research, Shandong University, Shandong Provincial Key New Think Tank, Jinan, China.

[4]Zhucheng Shiqiaozi Health Hospital, Zhucheng, China.

[5]Health Economics and Decision Science (HEDS), School of Health and Related Research (ScHARR), University of Sheffield, Sheffield, UK.

[6]China National Health Development Research Center, Beijing, China.

***Correspondence to:** Qiang Sun, qiangs@sdu.edu.cn & Jia Yin, yinjia@sdu.edu.cn

¶ Both authors contributed equally to this paper.

Citation: Fan Z, Si X, Wang Z, et al. A systematic review and quality assessment of pharmacoeconomic publications for China compared to internationally: Is the quality of evidence-base sufficient for health technology assessment? Int J Health Policy Manag. 2025;14:8656. doi:10.34172/ijhpm.8656

Supplementary file 1

1. Search Strategies

1.1 Search Strategies of Systematic Review

Table 1. Search Strategies of Systematic Review

	Search Strategies
#1	（pharmacoeconomics OR pharmacoeconomic evaluation OR health economic）[Title/Abstract]
#2	（minimum cost OR cost-effectiveness OR cost-benefit OR cost-utility）[Title/Abstract]
#3	#1 OR #2
#4	（China OR Chinese）[Title/Abstract]
#5	#3 AND #4

1.2 Search Strategies of Umbrella Review

Table 2. Search Strategies of Umbrella Review

	Search Strategies
#1	(pharmacoeconomics OR pharmacoeconomic evaluation OR health economics)[All fields]
#2	(minimum cost OR cost-effectiveness OR cost-benefit OR cost-utility)[All fields]
#3	#1 OR #2
#4	(systematic review OR review)[Title]
#5	(quality OR quality evaluation OR quality assessment OR assessment of quality)[Title]
#6	#4 OR #5
#7	#3 AND #6

2. Basic information of studies included systematic review

Table 3. Basic information of studies included systematic review.

Num.	Study	Year of publication	Chinese /English	Affiliation of author	journal level	Distribution of diseases	Perspective	Type of analysis	Model	Time horizon	Types of costs	Discount rate	Outcomes	Incremental analysis (Yes/No)	Threshold	Sensitivity analysis
1	Chen et al.	2018	Chinese	Medical institutions	National journal	Tumor	Not reported	CEA	Markov model	Long-term	Not specified	Not reported	Clinical Efficacy/ effectiveness /utility	NO	Not reported	One-way sensitivity analysis
2	Yu et al.	2018	Chinese	Medical institutions	Core journal	Tumor	Not reported	CEA	Not used	Short-term	Direct costs	Not reported	Clinical Efficacy/ effectiveness /utility	NO	Not reported	Not specified
3	Xie et al.	2018	Chinese	Medical institutions	Core journal	Cardiovascular and cerebrovascular diseases	Not reported	CEA	Decision tree model	Long-term	Direct costs	Not reported	Utility and clinical efficacy/effectiveness	Yes	1-3 times per capita GDP	One-way sensitivity analysis
4	Long et al.	2018	Chinese	Medical institutions	Core journal	Infectious diseases	Not reported	CEA	Not used	Short-term	Not specified	Not reported	Clinical Efficacy/ effectiveness /utility	NO	Not reported	Not specified
5	Qin et al.	2018	Chinese	Medical institutions	Core journal	Tumor	Healthcare system	CEA	Markov model	Short-term	Direct medical costs	Not reported	Utility and clinical efficacy/effectiveness	Yes	1-3 times per capita GDP	Use both of the above analyses
6	Chen et al.	2018	Chinese	Medical institutions	Core journal	Tumor	Not reported	CEA	Not used	Long-term	Direct costs	Not reported	Clinical Efficacy/ effectiveness /utility	NO	Not reported	Not specified
7	Xie et al.	2018	Chinese	Medical institutions	Core journal	Tumor	Not reported	CEA	Not used	Long-term	Direct costs	Not reported	Clinical Efficacy/ effectiveness /utility	NO	Not reported	Not specified
8	Xu et al.	2018	Chinese	Medical institutions	Core journal	Tumor	Not reported	CEA	Not used	Short-term	Direct costs	Not reported	Clinical efficacy/ effectiveness and Incidence of adverse reactions	NO	Not reported	Not specified

9	Wang et al.	2018	Chinese	Medical institutions	National journal	Digestive system diseases	Not reported	CEA	Not used	Long-term	Direct costs	Not reported	Utility and clinical efficacy/effectiveness	Yes	Not reported	Not specified
10	Hu et al.	2018	Chinese	Medical institutions	National journal	Tumor	Not reported	CEA	Not used	Long-term	Direct medical costs	Not reported	Utility and clinical efficacy/effectiveness	NO	Not reported	Not specified
11	Tian et al.	2018	Chinese	Medical institutions	National journal	Digestive system diseases	Healthcare system	CEA	Decision tree model	Short-term	Direct costs	Other	Clinical Efficacy/ effectiveness /utility	Yes	1-3 times per capita GDP	One-way sensitivity analysis
12	Yuan et al.	2019	Chinese	Medical institutions	National journal	Disease of respiratory system	Not reported	CEA	Not used	Short-term	Not specified	Not reported	Clinical Efficacy/ effectiveness /utility	NO	Not reported	Not specified
13	Xu et al.	2022	Chinese	Universities	National journal	Digestive system diseases	Not reported	Use both of the above analyses	Not used	Not specified	Direct costs	Other	Clinical Efficacy/ effectiveness /utility	Yes	1-3 times per capita GDP	Use both of the above analyses
14	Wei et al.	2022	Chinese	Universities and medical institutions	National journal	Urogenital diseases	Healthcare system	CEA	Not used	Not specified	Direct costs	Not reported	Clinical Efficacy/ effectiveness /utility	Yes	Other	One-way sensitivity analysis
15	Chen et al.	2022	Chinese	Other	National journal	Infectious diseases	Not reported	CEA	Not used	Not specified	Direct and indirect costs	Not reported	Utility and clinical efficacy/effectiveness	Yes	Not reported	Not specified
16	Lu et al.	2022	Chinese	Other	National journal	Infectious diseases	Payer	CEA	Not used	Not specified	Direct costs	Not reported	Utility and clinical efficacy/effectiveness	Yes	1-3 times per capita GDP	Use both of the above analyses
17	Jing et al.	2022	Chinese	Universities	Core journal	Cardiovascular and cerebrovascular diseases	Not reported	CEA	Markov model	Not specified	Direct and indirect costs	5%	Clinical Efficacy/ effectiveness /utility	NO	Not reported	Use both of the above analyses
18	Wang et al.	2022	Chinese	Medical institutions	National journal	Infectious diseases	Health care provider	CEA	Not used	Not specified	Direct medical costs	Not reported	Clinical Efficacy/ effectiveness /utility	NO	Not reported	One-way sensitivity analysis
19	Ma et al.	2022	Chinese	Medical institutions	National	Cardiovascular	Not reported	Use both	Not used	Not	Direct costs	Not	Clinical Efficacy/	Yes	1-3 times per	One-way sensitivity

					journal	and cerebrovascular diseases		of the above analyses		specified		reported	effectiveness /utility		capita GDP	analysis
20	Zhang et al.	2021	Chinese	Universities and medical institutions	National journal	Else	Not reported	CEA	Decision tree model	Not specified	Direct and indirect costs	Not reported	Utility and clinical efficacy/effectiveness	Yes	Not reported	Not specified
21	Jing et al.	2021	Chinese	Universities	National journal	Cardiovascular and cerebrovascular diseases	Not reported	Use both of the above analyses	Not used	Not specified	Direct costs	Not reported	Utility and clinical efficacy/effectiveness	Yes	Not reported	Not specified
22	Zhang et al.	2020	Chinese	Other	National journal	Digestive system diseases	Health care provider	Use both of the above analyses	Not used	Not specified	Direct costs	Not reported	Utility and clinical efficacy/effectiveness	Yes	Not reported	One-way sensitivity analysis
23	Chen et al.	2018	Chinese	Other	National journal	Cardiovascular and cerebrovascular diseases	Not reported	CEA	Not used	Not specified	Direct costs	Not reported	Utility and clinical efficacy/effectiveness	Yes	1-3 times per capita GDP	One-way sensitivity analysis
24	He et al.	2020	Chinese	Medical institutions	National journal	Diabetes	Not reported	CEA	Not used	Long-term	Direct medical costs	Not reported	Clinical Efficacy/ effectiveness /utility	Yes	Not reported	One-way sensitivity analysis
25	Qi et al.	2020	Chinese	Medical institutions	Core journal	Tumor	Not reported	CEA	Not used	Long-term	Direct medical costs	Not reported	Clinical Efficacy/ effectiveness /utility	NO	Not reported	Not specified
26	Jin et al.	2020	Chinese	Universities and medical institutions	National journal	Cardiovascular and cerebrovascular diseases	Health care provider	CEA	Not used	Long-term	Direct costs	Not reported	Utility and clinical efficacy/effectiveness	Yes	Not reported	One-way sensitivity analysis
27	Zhang et al.	2020	Chinese	Universities and	Core	Tumor	Healthcare	CEA	Markov model	Not	Direct medical	3%	Utility and clinical	Yes	1-3 times per	Use both of the

				medical institutions	journal		system			specified	costs		efficacy/effectiveness		capita GDP	above analyses
28	Yin et al.	2020	Chinese	Medical institutions	National journal	Disease of respiratory system	Not reported	CEA	Not used	Short-term	Direct medical costs	Not reported	Clinical Efficacy/ effectiveness /utility	NO	Not reported	One-way sensitivity analysis
29	Ren et al.	2020	Chinese	Medical institutions	Core journal	Tumor	Not reported	Use both of the above analyses	Not used	Long-term	Direct medical costs	Not reported	Utility and clinical efficacy/effectiveness	Yes	Not reported	Other
30	Lu et al.	2021	Chinese	Medical institutions	National journal	Digestive system diseases	Not reported	CEA	Not used	Long-term	Not specified	Not reported	Clinical Efficacy/ effectiveness /utility	NO	Not reported	One-way sensitivity analysis
31	Tao et al.	2021	Chinese	Medical institutions	Core journal	Diabetes	Not reported	CEA	Not used	Long-term	Direct costs	Not reported	Clinical Efficacy/ effectiveness /utility	NO	Not reported	Not specified
32	Xu et al.	2021	Chinese	Medical institutions	Core journal	Urogenital diseases	Health care provider	CEA	Decision tree model	Short-term	Direct medical costs	Not reported	Clinical Efficacy/ effectiveness /utility	Yes	Not reported	Use both of the above analyses
33	Liu et al.	2021	Chinese	Medical institutions	National journal	Infectious diseases	Health care provider	CEA	Decision tree model	Short-term	Direct medical costs	Other	Clinical Efficacy/ effectiveness /utility	Yes	Not reported	Use both of the above analyses
34	Wang et al.	2021	Chinese	Medical institutions	National journal	Cardiovascular and cerebrovascular diseases	Patient	CMA	Not used	Long-term	Direct costs	Other	Clinical Efficacy/ effectiveness /utility	NO	Not reported	Other
35	Zhang et al.	2021	Chinese	Medical institutions	Core journal	Else	Not reported	Use both of the above analyses	Decision tree model	Long-term	Direct costs	Other	Clinical efficacy/ effectiveness and Incidence of adverse reactions	NO	Not reported	One-way sensitivity analysis
36	Chen et al.	2021	Chinese	Medical institutions	Core journal	Infectious diseases	Not reported	CEA	Not used	Long-term	Direct medical costs	Other	Clinical Efficacy/ effectiveness /utility	Yes	1-3 times per capita GDP	One-way sensitivity analysis

37	Yang et al.	2019	Chinese	Universities	Core journal	Infectious diseases	Healthcare system	CEA	Not used	Long-term	Direct medical costs	Other	Clinical Efficacy/ effectiveness /utility	Yes	1-3 times per capita GDP	Use both of the above analyses
38	Yang et al.	2021	Chinese	Universities and medical institutions	National journal	Else	Societal	CEA	Not used	Long-term	Direct and indirect costs	Other	Clinical Efficacy/ effectiveness /utility	Yes	Not reported	One-way sensitivity analysis
39	Ma et al.	2018	Chinese	Universities	National journal	Else	Societal	CUA	Markov model	Long-term	Direct medical costs	3%	Clinical Efficacy/ effectiveness /utility	Yes	Other	Use both of the above analyses
40	Fu et al.	2019	Chinese	Universities	Core journal	Else	Healthcare system	Use both of the above analyses	Not used	Short-term	Direct medical costs	Other	Clinical Efficacy/ effectiveness /utility	Yes	Not reported	Use both of the above analyses
41	Xiong et al.	2019	Chinese	Universities	Core journal	Urogenital diseases	Patient	CUA	Markov model	Long-term	Direct medical costs	5%	Clinical Efficacy/ effectiveness /utility	Yes	Other	Use both of the above analyses
42	Chen et al.	2020	Chinese	Universities	Core journal	Infectious diseases	Not reported	CEA	Markov model	Long-term	Direct medical costs	5%	Clinical Efficacy/ effectiveness /utility	Yes	Other	Use both of the above analyses
43	Xi et al.	2020	Chinese	Universities	Core journal	Diabetes	Not reported	CEA	Markov model	Long-term	Direct medical costs	3%	Clinical Efficacy/ effectiveness /utility	Yes	1-3 times per capita GDP	Use both of the above analyses
44	Liu et al.	2020	Chinese	Universities	National journal	Else	Not reported	CMA	Not used	Long-term	Direct medical costs	Not reported	Clinical Efficacy/ effectiveness /utility	NO	Not reported	Not specified
45	Li et al.	2021	Chinese	Universities	National journal	Tumor	Health care provider	CEA	Partitioned survival model	Long-term	Direct medical costs	5%	Clinical Efficacy/ effectiveness /utility	Yes	1-3 times per capita GDP	Use both of the above analyses
46	Wei et al.	2021	Chinese	Universities	Core journal	Infectious diseases	Societal	CUA	Markov model	Long-term	Direct and indirect costs	5%	Clinical Efficacy/ effectiveness /utility	Yes	1-3 times per capita GDP	Use both of the above analyses
47	Li et al.	2021	Chinese	Universities and medical institutions	National journal	Cardiovascular and cerebrovascular diseases	Not reported	Use both of the above analyses	Not used	Long-term	Direct medical costs	5%	Clinical Efficacy/ effectiveness /utility	Yes	1-3 times per capita GDP	Use both of the above analyses

48	Wang et al.	2021	Chinese	Universities and medical institutions	Core journal	Cardiovascular and cerebrovascular diseases	Payer	CEA	Markov model	Long-term	Direct medical costs	5%	Clinical Efficacy/ effectiveness /utility	Yes	1-3 times per capita GDP	Use both of the above analyses
49	Zhou et al.	2022	Chinese	Universities	Core journal	Tumor	Healthcare system	CEA	Markov model	Long-term	Direct medical costs	5%	Clinical Efficacy/ effectiveness /utility	Yes	1-3 times per capita GDP	Use both of the above analyses
50	Zuo et al.	2020	Chinese	Universities	National journal	Diabetes	Not reported	CEA	Not used	Not specified	Direct medical costs	Not reported	Utility and clinical efficacy/effectiveness	Yes	Not reported	One-way sensitivity analysis
51	Xu et al.	2022	English	Universities	Q1	Else	Payer	CEA	Decision tree model	Short-term	Direct medical costs	Other	Clinical Efficacy/ effectiveness /utility	Yes	Other	Use both of the above analyses
52	Luo et al.	2022	English	Medical institutions	Q4	Tumor	Health care provider	CEA	Markov model	Long-term	Direct medical costs	5%	Clinical Efficacy/ effectiveness /utility	Yes	1-3 times per capita GDP	One-way sensitivity analysis
53	Liu et al.	2022	English	Medical institutions	Q1	Tumor	Payer	CEA	Other	Not specified	Direct medical costs	3%	Utility and clinical efficacy/effectiveness	Yes	Other	Use both of the above analyses
54	Gu et al.	2022	English	Medical institutions	Q1	Disease of respiratory system	Health care provider	CEA	Not used	Short-term	Direct medical costs	Other	Clinical Efficacy/ effectiveness /utility	Yes	Other	Other
55	Lu et al.	2022	English	Universities	Q3	Cardiovascular and cerebrovascular diseases	Patient	CEA	Decision tree model	Not specified	Direct medical costs	5%	Clinical Efficacy/ effectiveness /utility	Yes	Other	Use both of the above analyses
56	Hui et al.	2019	Chinese	Medical institutions	National journal	Else	Not reported	CEA	Not used	Not specified	Direct medical costs	Not reported	Clinical Efficacy/ effectiveness /utility	Yes	Not reported	Not specified
57	Chai et al.	2019	Chinese	Medical institutions	Core journal	Else	Not reported	CEA	Decision tree model	Short-term	Direct medical costs	Not reported	Utility and clinical efficacy/effectiveness	Yes	Not reported	Use both of the above analyses
58	Li et al.	2019	Chinese	Medical institutions	National	Else	Not reported	CMA	Not used	Short-term	Direct costs	Other	Clinical efficacy/	NO	Not reported	One-way sensitivity

					journal								effectiveness and Incidence of adverse reactions			analysis
59	Si et al.	2019	Chinese	Medical institutions	National journal	Tumor	Not reported	CEA	Not used	Long-term	Direct medical costs	Not reported	Utility and clinical efficacy/effectiveness	Yes	Not reported	One-way sensitivity analysis
60	Wang et al.	2019	Chinese	Medical institutions	National journal	Cardiovascular and cerebrovascular diseases	Health care provider	CEA	Not used	Short-term	Direct medical costs	Not reported	Utility and clinical efficacy/effectiveness	Yes	1-3 times per capita GDP	One-way sensitivity analysis
61	Xie et al.	2019	Chinese	Medical institutions	Core journal	Tumor	Not reported	CEA	Not used	Long-term	Direct medical costs	Not reported	Clinical Efficacy/ effectiveness /utility	Yes	Not reported	Not specified
62	Chen et al.	2019	Chinese	Medical institutions	Core journal	Infectious diseases	Health care provider	CMA	Not used	Long-term	Direct medical costs	Other	Clinical Efficacy/ effectiveness /utility	NO	Not reported	Not specified
63	Chen et al.	2020	Chinese	Medical institutions	National journal	Infectious diseases	Not reported	CEA	Not used	Long-term	Direct medical costs	Not reported	Utility and clinical efficacy/effectiveness	Yes	Not reported	One-way sensitivity analysis
64	Zhan et al.	2020	Chinese	Medical institutions	National journal	Infectious diseases	Not reported	CEA	Not used	Long-term	Direct costs	Other	Utility and clinical efficacy/effectiveness	Yes	Not reported	One-way sensitivity analysis
65	Liu et al.	2020	Chinese	Medical institutions	National journal	Cardiovascular and cerebrovascular diseases	Not reported	CEA	Not used	Long-term	Direct costs	Not reported	Clinical Efficacy/ effectiveness /utility	NO	Not reported	One-way sensitivity analysis
66	Yan et al.	2020	Chinese	Medical institutions	National journal	Infectious diseases	Not reported	CEA	Not used	Short-term	Direct medical costs	Not reported	Utility and clinical efficacy/effectiveness	Yes	Not reported	One-way sensitivity analysis
67	Guan et al.	2019	English	Universities	Q3	Tumor	Healthcare system	CEA	Markov model	Long-term	Direct medical costs	3%	Utility and clinical efficacy/effectiveness	Yes	Other	Use both of the above analyses
68	Jiang et al.	2021	English	Universities	Q3	Infectious	Health care	CEA	Decision tree	Short-term	Direct medical	5%	Clinical Efficacy/	Yes	1-3 times per	Use both of the

						diseases	provider		model		costs		effectiveness /utility		capita GDP	above analyses
69	Gao et al.	2021	English	Universities	Q3	Tumor	Healthcare system	CBA	Partitioned survival model	Long-term	Direct medical costs	5%	Utility and clinical efficacy/effectiveness	Yes	1-3 times per capita GDP	Use both of the above analyses
70	Yang et al.	2020	English	Other	Q3	Else	Payer	CUA	Markov model	Long-term	Direct medical costs	5%	Clinical Efficacy/ effectiveness /utility	Yes	Not reported	Use both of the above analyses
71	Hou et al.	2019	English	Medical institutions	Q2	Diabetes	Health care provider	CEA	Not used	Not specified	Direct medical costs	5%	Clinical Efficacy/ effectiveness /utility	Yes	1-3 times per capita GDP	Use both of the above analyses
72	Cheng et al.	2019	English	Medical institutions	Q3	Diabetes	Healthcare system	CBA	Not used	Not specified	Direct medical costs	5%	Clinical Efficacy/ effectiveness /utility	Yes	Other	Use both of the above analyses
73	Rui et al.	2021	English	Universities	Q3	Else	Patient	CUA	Markov model	Long-term	Direct medical costs	Other	Clinical Efficacy/ effectiveness /utility	Yes	1-3 times per capita GDP	Use both of the above analyses
74	Wang et al.	2021	English	Universities	Q3	Tumor	Healthcare system	CBA	Partitioned survival model	Long-term	Direct medical costs	3%	Utility and clinical efficacy/effectiveness	Yes	Other	Use both of the above analyses
75	Zhou et al.	2022	English	Universities	Q3	Tumor	Societal	CUA	Partitioned survival model	Long-term	Direct medical costs	5%	Utility and clinical efficacy/effectiveness	Yes	Other	Use both of the above analyses
76	Wang et al.	2020	English	Other	Core journal	Else	Not reported	CUA	Markov model	Not specified	Direct costs	Other	Utility and clinical efficacy/effectiveness	Yes	Other	Use both of the above analyses
77	Tian et al.	2018	Chinese	Universities	Core journal	Cardiovascular and cerebrovascular diseases	Patient	CEA	Not used	Not specified	Direct medical costs	Other	Clinical Efficacy/ effectiveness /utility	Yes	Other	One-way sensitivity analysis
78	Zhou et al.	2018	Chinese	Universities and medical institutions	National journal	Else	Societal	CEA	Not used	Short-term	Direct costs	Other	Clinical Efficacy/ effectiveness /utility	Yes	Other	One-way sensitivity analysis
79	Qi et al.	2018	Chinese	Universities and medical institutions	Core journal	Else	Not reported	Use both of the above	Not used	Short-term	Direct medical costs	Not reported	Clinical Efficacy/ effectiveness /utility	NO	Not reported	One-way sensitivity analysis

								analyses								
80	Tang et al.	2018	Chinese	Universities	Core journal	Tumor	Patient	CUA	Markov model	Long-term	Direct medical costs	5%	Clinical Efficacy/ effectiveness /utility	Yes	1-3 times per capita GDP	Use both of the above analyses
81	Zhang et al.	2018	Chinese	Universities and medical institutions	National journal	Infectious diseases	Health care provider	CEA	Decision tree model	Not specified	Direct costs	Not reported	Clinical Efficacy/ effectiveness /utility	Yes	Not reported	One-way sensitivity analysis
82	Yang et al.	2018	Chinese	Universities	Core journal	Diabetes	Payer	CEA	Decision tree model	Not specified	Direct medical costs	Other	Clinical Efficacy/ effectiveness /utility	NO	Not reported	One-way sensitivity analysis
83	Xuan et al.	2018	English	Other	Q3	Cardiovascular and cerebrovascular diseases	Payer	CUA	Decision tree model	Long-term	Direct medical costs	3%	Utility and clinical efficacy/effectiveness	Yes	1-3 times per capita GDP	Use both of the above analyses
84	Wang et al.	2018	English	Medical institutions	Q4	Tumor	Health care provider	CEA	Markov model	Long-term	Direct medical costs	Other	Clinical Efficacy/ effectiveness /utility	Yes	1-3 times per capita GDP	Use both of the above analyses
85	Zhou et al.	2019	English	Universities	Q3	Else	Societal	CEA	Decision tree model	Short-term	Direct medical costs	Other	Clinical Efficacy/ effectiveness /utility	Yes	Other	One-way sensitivity analysis
86	Wang et al.	2019	English	Medical institutions	Q1	Diabetes	Health care provider	CEA	Not used	Short-term	Direct medical costs	Other	Clinical Efficacy/ effectiveness /utility	Yes	1-3 times per capita GDP	One-way sensitivity analysis
87	Zhang et al.	2019	English	Medical institutions	Q1	Infectious diseases	Patient	CEA	Decision tree model	Not specified	Direct medical costs	Not reported	Clinical Efficacy/ effectiveness /utility	Yes	Other	Use both of the above analyses
88	Zhou et al.	2019	English	Medical institutions	Q1	Tumor	Not reported	CEA	Decision tree model	Not specified	Direct medical costs	Other	Clinical Efficacy/ effectiveness /utility	Yes	1-3 times per capita GDP	Use both of the above analyses
89	Liu et al.	2020	English	Medical institutions	Q2	Else	Healthcare system	CUA	Markov model	Long-term	Direct medical costs	5%	Clinical Efficacy/ effectiveness /utility	Yes	Other	One-way sensitivity analysis
90	Chen et al.	2022	Chinese	Medical institutions	Core journal	Cardiovascular and cerebrovascular	Health care provider	CUA	Markov model	Long-term	Direct medical costs	5%	Clinical Efficacy/ effectiveness /utility	Yes	1-3 times per capita GDP	Use both of the above analyses

						diseases										
91	Tian et al.	2020	English	Universities	Q1	Else	Healthcare system	CEA	Markov model	Long-term	Direct medical costs	5%	Clinical Efficacy/ effectiveness /utility	Yes	Other	Use both of the above analyses
92	Zeng et al.	2021	English	Medical institutions	Q4	Else	Not reported	CEA	Markov model	Short-term	Direct medical costs	Other	Clinical Efficacy/ effectiveness /utility	Yes	1-3 times per capita GDP	Use both of the above analyses
93	Cui et al.	2021	English	Universities	Q2	Else	Patient	CEA	Markov model	Long-term	Direct costs	Other	Clinical Efficacy/ effectiveness /utility	Yes	1-3 times per capita GDP	Use both of the above analyses
94	Sun et al.	2021	English	Universities	Q3	Cardiovascular and cerebrovascular diseases	Healthcare system	CMA	Not used	Long-term	Direct costs	Not reported	Clinical Efficacy/ effectiveness /utility	NO	Not reported	One-way sensitivity analysis
95	Bao et al.	2021	English	Medical institutions	Q3	Tumor	Healthcare system	CEA	Markov model	Long-term	Direct costs	3%	Clinical Efficacy/ effectiveness /utility	Yes	1-3 times per capita GDP	Use both of the above analyses
96	Jiang et al.	2022	English	Universities	Q4	Tumor	Healthcare system	CEA	Markov model	Long-term	Direct costs	3%	Clinical Efficacy/ effectiveness /utility	Yes	1-3 times per capita GDP	Use both of the above analyses
97	Jia et al.	2019	Chinese	Other	National journal	Tumor	Patient	CUA	Markov model	Long-term	Direct costs	5%	Clinical Efficacy/ effectiveness /utility	Yes	Other	Use both of the above analyses
98	Mu et al.	2019	Chinese	Universities	National journal	Digestive system diseases	Healthcare system	CEA	Decision tree model	Short-term	Direct costs	Not reported	Clinical Efficacy/ effectiveness /utility	Yes	Other	One-way sensitivity analysis
99	Liu et al.	2020	Chinese	Universities	Core journal	Cardiovascular and cerebrovascular diseases	Not reported	CEA	Not used	Not specified	Direct costs	Not reported	Clinical efficacy/ effectiveness and Incidence of adverse reactions	Yes	Other	Use both of the above analyses
100	Shi et al.	2020	Chinese	Universities	Core journal	Infectious diseases	Not reported	CEA	Decision tree model	Not specified	Direct medical costs	Not reported	Clinical efficacy/ effectiveness and Incidence of adverse	Yes	Not reported	One-way sensitivity analysis

													reactions			
101	Wang et al.	2020	Chinese	Universities	National journal	Cardiovascular and cerebrovascular diseases	Healthcare system	CMA	Decision tree model	Short-term	Direct medical costs	Not reported	Clinical Efficacy/ effectiveness /utility	NO	Not reported	Not specified
102	Liu et al.	2021	Chinese	Medical institutions	Core journal	Tumor	Healthcare system	CUA	Partitioned survival model	Long-term	Direct costs	5%	Clinical Efficacy/ effectiveness /utility	Yes	1-3 times per capita GDP	Use both of the above analyses
103	Shi et al.	2022	Chinese	Universities and medical institutions	Core journal	Tumor	Healthcare system	CUA	Markov model	Long-term	Direct costs	5%	Clinical Efficacy/ effectiveness /utility	Yes	1-3 times per capita GDP	Use both of the above analyses
104	Wu et al.	2021	Chinese	Medical institutions	National journal	Cardiovascular and cerebrovascular diseases	Patient	CUA	Markov model	Long-term	Direct costs	3%	Clinical Efficacy/ effectiveness /utility	Yes	1-3 times per capita GDP	Use both of the above analyses
105	Yang et al.	2021	Chinese	Medical institutions	Core journal	Infectious diseases	Healthcare system	CEA	Not used	Long-term	Direct medical costs	5%	Clinical Efficacy/ effectiveness /utility	Yes	1-3 times per capita GDP	One-way sensitivity analysis
106	Gong et al.	2022	Chinese	Medical institutions	National journal	Cardiovascular and cerebrovascular diseases	Societal	CUA	Markov model	Long-term	Direct costs	5%	Clinical Efficacy/ effectiveness /utility	Yes	1-3 times per capita GDP	Use both of the above analyses
107	Pang et al.	2022	Chinese	Medical institutions	Core journal	Else	Not reported	CEA	Not used	Not specified	Direct medical costs	Not reported	Utility and clinical efficacy/effectiveness	Yes	Not reported	One-way sensitivity analysis
108	Peng et al.	2022	Chinese	Medical institutions	Core journal	Digestive system diseases	Not reported	CMA	Not used	Not specified	Direct costs	Not reported	Clinical Efficacy/ effectiveness /utility	Yes	Not reported	One-way sensitivity analysis
109	Pei et al.	2022	Chinese	Medical institutions	Core journal	Digestive system diseases	Not reported	CMA	Not used	Not specified	Direct costs	Not reported	Clinical efficacy/ effectiveness and Incidence of adverse	NO	Not reported	One-way sensitivity analysis

													reactions			
110	Zhang et al.	2022	Chinese	Medical institutions	Core journal	Tumor	Healthcare system	CUA	Markov model	Long-term	Direct costs	5%	Clinical Efficacy/ effectiveness /utility	Yes	1-3 times per capita GDP	Use both of the above analyses
111	Qi et al.	2022	Chinese	Medical institutions	Core journal	Tumor	Healthcare system	CUA	Partitioned survival model	Long-term	Direct costs	5%	Clinical Efficacy/ effectiveness /utility	Yes	Other	Use both of the above analyses
112	Li et al.	2022	Chinese	Medical institutions	Core journal	Tumor	Healthcare system	CEA	Partitioned survival model	Short-term	Direct costs	5%	Clinical Efficacy/ effectiveness /utility	Yes	1-3 times per capita GDP	Use both of the above analyses
113	Gao et al.	2022	Chinese	Medical institutions	National journal	Cardiovascular and cerebrovascular diseases	Healthcare system	CEA	Decision tree model	Short-term	Direct costs	Not reported	Clinical Efficacy/ effectiveness /utility	Yes	Other	Use both of the above analyses
114	Dai et al.	2023	English	Universities	Q2	Tumor	Healthcare system	CEA	Partitioned survival model	Long-term	Direct medical costs	5%	effectiveness	Yes	1-3 times per capita GDP	Use both of the above analyses
115	Fei et al.	2023	English	Universities	Q3	Tumor	Healthcare system	CEA	Markov model	Long-term	Direct medical costs	5%	effectiveness	Yes	WTP	Use both of the above analyses
116	Gong et al.	2023	English	Universities	Q3	Tumor	Patient	CEA	Partitioned survival model	Long-term	Direct costs	5%	effectiveness	Yes	WTP	Use both of the above analyses
117	Hu et al.	2023	English	Universities and medical institutions	Q1	Diabetes	Health care provider	CUA	Other	Long-term	Direct medical costs	5%	Utility	Yes	WTP	Use both of the above analyses
118	Hu et al.	2023	English	Medical institutions	Q3	Cardiovascular and cerebrovascular diseases	Healthcare system	CEA	Markov model	Short-term	Direct costs	3%	effectiveness	Yes	1-3 times per capita GDP	Use both of the above analyses
119	Huang et al.	2023	English	Universities and medical institutions	Q3	Tumor	Health care provider	CEA	Markov model	Long-term	Direct medical costs	Not reported	effectiveness	Yes	WTP	Use both of the above analyses
120	Huo et al.	2023	English	Universities and	Q1	Tumor	Payer	CEA	Markov model	Long-term	Direct medical	5%	effectiveness	Yes	WTP	Use both of the the

				medical institutions							costs					above analyses
121	Kong et al.	2023	English	Medical institutions	Q1	Else	Payer	CEA	Use both of the above models	Long-term	Direct medical costs	5%	effectiveness	Yes	WTP	Use both of the above analyses
122	Li et al.	2023	English	Universities and medical institutions	Q2	Tumor	Healthcare system	CEA	Use both of the above models	Long-term	Direct medical costs	5%	effectiveness and Utility	Yes	WTP	Use both of the above analyses
123	Chen et al.	2023	Chinese	Other	Core journal	Else	Societal	Use both of the above analyses	Partitioned survival model	Short-term	Direct and indirect costs	Other	effectiveness	Yes	Other	Not specified
124	Li et al.	2023	English	Medical institutions	Q1	Tumor	Healthcare system	CUA	Use both of the above models	Long-term	Direct medical costs	5%	Utility	Yes	WTP	Use both of the above analyses
125	Li et al.	2023	English	Medical institutions	Q3	Disease of respiratory system	Patient	CUA	Markov model	Long-term	Direct medical costs	5%	Utility	Yes	1-3 times per capita GDP	Use both of the above analyses
126	Liang et al.	2023	English	Universities and medical institutions	Q3	Tumor	Healthcare system	CUA	Partitioned survival model	Long-term	Direct medical costs	5%	Utility	Yes	1-3 times per capita GDP	Use both of the above analyses
127	Lin et al.	2023	English	Universities	Q3	Tumor	Healthcare system	CUA	Use both of the above models	Long-term	Direct medical costs	5%	Utility	Yes	1-3 times per capita GDP	Use both of the above analyses
128	Liu et al.	2023	English	Universities and medical institutions	Q3	Diabetes	Healthcare system	CUA	Other	Long-term	Direct medical costs	5%	Utility	Yes	1-3 times per capita GDP	Use both of the above analyses
129	Liu et al.	2023	English	Universities	Q1	Tumor	Healthcare system	CUA	Partitioned survival model	Long-term	Direct medical costs	5%	Utility	Yes	1-3 times per capita GDP	Use both of the above analyses

130	Liu et al.	2023	English	Universities	Q1	Tumor	Healthcare system	CUA	Partitioned survival model	Long-term	Direct medical costs	5%	Utility	Yes	1-3 times per capita GDP	Use both of the above analyses
131	Nie et al.	2023	English	Universities and medical institutions	Q1	Tumor	Healthcare system	CUA	Partitioned survival model	Long-term	Direct medical costs	5%	Utility	Yes	1-3 times per capita GDP	Use both of the above analyses
132	Qiu et al.	2023	English	Universities	Q1	Tumor	Healthcare system	CUA	Partitioned survival model	Long-term	Direct medical costs	5%	Utility	Yes	1-3 times per capita GDP	Use both of the above analyses
133	Shang et al.	2023	English	Universities and medical institutions	Q3	Tumor	Healthcare system	CUA	Partitioned survival model	Long-term	Direct medical costs	5%	Utility	Yes	1-3 times per capita GDP	Use both of the above analyses
134	Shao et al.	2023	English	Universities	Q1	Tumor	Not reported	CUA	Partitioned survival model	Long-term	Direct medical costs	Not reported	Utility	Yes	1-3 times per capita GDP	Use both of the above analyses
135	Shi et al.	2023	English	Medical institutions	Q1	Infectious diseases	Healthcare system	CEA	Decision tree model	Long-term	Direct medical costs	Not reported	effectiveness	Yes	Not reported	Use both of the above analyses
136	Shi et al.	2023	English	Medical institutions	Q4	Tumor	Healthcare system	CEA	Partitioned survival model	Long-term	Direct costs	3%	effectiveness	Yes	WTP	Use both of the above analyses
137	Shu et al.	2023	English	Medical institutions	Q2	Tumor	Healthcare system	CEA	Markov model	Long-term	Direct medical costs	3%	effectiveness	Yes	WTP	Use both of the above analyses
138	Shu et al.	2023	English	Medical institutions	Q2	Tumor	Healthcare system	CEA	Partitioned survival model	Long-term	Direct medical costs	5%	effectiveness	Yes	1-3 times per capita GDP	Use both of the above analyses
139	Wang et al.	2023	English	Medical institutions	Q2	Tumor	Payer	CEA	Markov model	Long-term	Direct medical costs	5%	effectiveness	Yes	1-3 times per capita GDP	Use both of the above analyses
140	Wu et al..	2023	English	Universities	Q2	Tumor	Healthcare system	CEA	Markov model	Long-term	Direct medical costs	5%	effectiveness	Yes	1-3 times per capita GDP	Use both of the above analyses
141	Xu et al.	2023	English	Universities	Q4	Tumor	Healthcare system	CEA	Partitioned survival model	Long-term	Direct medical costs	5%	effectiveness	Yes	1-3 times per capita GDP	Use both of the above analyses
142	Ye et al.	2023	English	Universities and medical institutions	Q1	Cardiovascular and	Payer	CEA	Use both of the above	Short-term	Not specified	5%	effectiveness	Yes	WTP	Use both of the above analyses

						cerebrovascular diseases			models							
143	Lang et al.	2023	Chinese	Medical institutions	Core journal	Cardiovascular and cerebrovascular diseases	Healthcare system	CUA	Markov model	Long-term	Direct medical costs	5%	effectiveness and Utility	Yes	1-3 times per capita GDP	Use both of the above analyses
144	Qi et al.	2023	Chinese	Medical institutions	Core journal	Tumor	Healthcare system	CEA	Partitioned survival model	Long-term	Direct medical costs	5%	effectiveness	Yes	1-3 times per capita GDP	Use both of the above analyses
145	Rao et al.	2023	Chinese	Medical institutions	Core journal	Disease of respiratory system	Healthcare system	CEA	Markov model	Long-term	Direct medical costs	5%	effectiveness	Yes	Not reported	Use both of the above analyses
146	Ye et al.	2023	English	Medical institutions	Q1	Tumor	Payer	Use both of the above analyses	Markov model	Long-term	Direct medical costs	5%	effectiveness and Utility	Yes	WTP	Use both of the above analyses
147	Zeng et al.	2023	English	Medical institutions	Q2	Tumor	Payer	CEA	Markov model	Long-term	Direct medical costs	5%	Utility	Yes	WTP	Not specified
148	Zhang et al.	2023	English	Universities	Q2	Tumor	Healthcare system	CEA	Use both of the above models	Long-term	Direct medical costs	5%	effectiveness and Utility	Yes	1-3 times per capita GDP	Use both of the above analyses
149	Zhang et al.	2023	English	Universities	Q1	Tumor	Healthcare system	CEA	Partitioned survival model	Long-term	Direct medical costs	5%	effectiveness and Utility	Yes	WTP	Use both of the above analyses
150	Zhao et al.	2023	English	Universities	Q1	Tumor	Healthcare system	CEA	Markov model	Long-term	Direct medical costs	5%	effectiveness and Utility	Yes	1-3 times per capita GDP	Use both of the above analyses
151	Zhou et al.	2023	English	Medical institutions	Q1	Tumor	Payer	CEA	Markov model	Long-term	Direct medical costs	3%	effectiveness and Utility	Yes	WTP	Use both of the above analyses

152	Zhu et al.	2023	English	Universities	Q2	Tumor	Healthcare system	CEA	Markov model	Long-term	Direct medical costs	5%	effectiveness and Utility	Yes	WTP	Use both of the above analyses
153	Zhu et al.	2023	English	Medical institutions	Q1	Disease of respiratory system	Healthcare system	CEA	Use both of the above models	Long-term	Direct medical costs	3%	Utility	Yes	WTP	Use both of the above analyses
154	Wang et al.	2023	Chinese	Universities	Core journal	Tumor	Healthcare system	CEA	Partitioned survival model	Long-term	Direct medical costs	5%	effectiveness and Utility	Yes	1-3 times per capita GDP	Use both of the above analyses
