# Supplementary material for: A Systematic Review and Quality Assessment of Pharmacoeconomic Publications for China Compared to Internationally: Is the Quality of Evidence-Base Sufficient for Health Technology Assessment?
Source: Int J Health Policy Manag. 2025 Apr 28;14:8656. doi: 10.34172/ijhpm.8656 (PMC12257205; doi:10.34172/ijhpm.8656)
Supplement: Supplementary file 2 — describes the CHEERS 2022 scores for the systematic review of the included studies. [file ijhpm-14-8656-s002.pdf]

Article title: A Systematic Review and Quality Assessment of Pharmacoeconomic Publications for China Compared to Internationally: Is the Quality of Evidence-Base Sufficient for Health Technology Assessment?

Journal name: International Journal of Health Policy and Management

Authors' information: Zhixin Fan[1,2,3]¶, Xu Si[1,2,3]¶, Zhongxiang Wang[4], Liwei Zhang[1,2,3], Junyang Liu[1,2,3], Qing He[1,2,3], Matthew Franklin[5], Qiang Sun[1,2,3,6]*, Jia Yin[1,2,3]*

[1]Department of Social Medicine and Health Management, School of Public Health, Cheeloo College of Medicine, Shandong University, Jinan, China.

[2]NHC Key Lab of Health Economics and Policy Research, Shandong University, Jinan, China.

[3]Center for Health Management and Policy Research, Shandong University, Shandong Provincial Key New Think Tank, Jinan, China.

[4]Zhucheng Shiqiaozi Health Hospital, Zhucheng, China.

[5]Health Economics and Decision Science (HEDS), School of Health and Related Research (ScHARR), University of Sheffield, Sheffield, UK.

[6]China National Health Development Research Center, Beijing, China.

***Correspondence to:** Qiang Sun, qiangs@sdu.edu.cn & Jia Yin, yinjia@sdu.edu.cn

¶ Both authors contributed equally to this paper.

Citation: Fan Z, Si X, Wang Z, et al. A systematic review and quality assessment of pharmacoeconomic publications for China compared to internationally: Is the quality of evidence-base sufficient for health technology assessment? Int J Health Policy Manag. 2025;14:8656. doi:10.34172/ijhpm.8656

Supplementary file 2

1. CHEERS 2022 scores for the systematic review of the included studies.

Table 1. CHEERS 2022 scores for the systematic review of the included studies.

**Num.**	**Study**	**CHEERS 2022 checklist**																												**Quality score**	
		**1**	**2**	**3**	**4**	**5**	**6**	**7**	**8**	**9**	**10**	**11**	**12**	**13**	**14**	**15**	**16**	**17**	**18**	**19**	**20**	**21**	**22**	**23**	**24**	**25**	**26**	**27**	**28**	**Score**	**Percentage**
1	Chen et al.	0.5	0.5	1	0	0	1	1	0	1	0	1	1	0.5	0	0	1	0	0	0	1	0	1	1	1	0	0	0	0	12.5	44.64%
2	Yu et al.	1	1	1	0	1	1	1	0	1	0	1	1	1	1	1	0	0	0	0	1	0	0.5	1	0.5	0	1	0	0	16	57.14%
3	Xie et al.	1	1	1	0	0	1	0.5	0	0	0	1	1	1	1	1	0	0	0	0	1	0	1	1	0.5	0	0	0	0	13	46.43%
4	Long et al.	1	0.5	1	0	1	1	1	0	0	0	1	1	1	0	0	0	0	0	0	0	0	0.5	1	0	0	0.5	1	0	11.5	41.07%
5	Qin et al.	1	1	1	0	0	1	1	0.5	0.5	0.5	1	1	1	1	0.5	0.5	0	0	0	1	0	0.5	1	1	0	1	1	0	17	60.71%
6	Chen et al.	0.5	0.5	1	0	1	1	0.5	0	0	0	1	1	1	1	0	0	0	0	0	1	0	1	1	1	0	0	1	0	13.5	48.21%
7	Xie et al.	1	1	1	0	0	1	1	0	0	0	1	1	0.5	0	0	0	0	0	0	0	0	0.5	1	0.5	0	0.5	0	0	10	35.71%
8	Xu et al.	0.5	0.5	1	0	0	1	0.5	0	0	0	1	1	1	1	0	0	0	0	0	1	0	1	1	1	0	0.5	0	0	12	42.86%
9	Wang et al.	1	1	1	0	1	1	1	0	0	0	1	1	1	0.5	0	0	0	0	0	1	0	1	1	0	0	0	1	0	13.5	48.21%
10	Hu et al.	0.5	0.5	1	0	1	1	0.5	0	0	0	1	1	1	1	1	0	0	0	0	1	0	1	1	0	0	0	1	0	13.5	48.21%
11	Tian et al.	0.5	0.5	1	0	0	1	0.5	0.5	1	1	1	1	1	1	0	0.5	0	0	0	1	0	0.5	1	0.5	0	1	0	0	14.5	51.79%
12	Yuan et al.	0.5	0.5	1	0	1	1	0.5	0	0	0	1	0.5	0.5	0	0	0	0	0	0	0	0	0.5	1	0	0	0	0	0	8	28.57%
13	Xu et al.	0.5	0.5	1	0	0.5	1	0.5	0	0	0	0.5	1	0.5	1	0.5	0	0	0	0	1	0	0.5	1	0.5	0	1	1	0	12.5	44.64%
14	Wei et al.	0.5	1	1	0	1	1	0.5	0.5	0.5	0	1	1	1	1	0	0	0	0	0	1	0	0.5	1	0.5	0	1	0	0	14	50.00%
15	Chen et al.	1	0.5	1	0	0	1	0.5	0	0	0	1	1	1	1	0.5	0.5	0	0	0	1	0	1	1	0.5	0	0	0	0	12.5	44.64%
16	Lu et al.	0.5	0.5	1	0	0	1	1	0.5	0	0	1	1	1	1	1	0	0	0	0	1	0	1	1	0.5	0	1	0	0	14	50.00%
17	Jing et al.	0.5	0.5	1	0	0	1	0.5	0.5	0	0.5	1	1	1	1	0.5	0.5	0.5	0	0	1	0	1	1	1	0	1	0	0	15	53.57%
18	Wang et al.	0.5	0.5	1	0	0	1	0.5	0.5	0	0	1	1	1	1	0.5	0	0	0	0	1	0	1	1	1	0	1	0	0	13.5	48.21%
19	Ma et al.	0.5	0.5	1	0	0.5	1	0.5	0	0	0	1	1	1	1	1	0	0	0	0	1	0	1	1	0.5	0	1	0	0	13.5	48.21%
20	Zhang et al.	1	1	1	0	0	1	1	0	0	0	1	1	1	1	1	0	0	0	0	1	0	1	1	0.5	0	0	0	0	13.5	48.21%

21	Jing et al.	0.5	0.5	1	0	0	1	0.5	0	0	0	1	1	1	1	0	0	0	0	0	1	0	1	1	0.5	0	1	0	1	13	46.43%
22	Zhang et al.	0.5	0.5	1	0	0	1	1	0.5	0	0	1	1	1	1	0.5	0	0	0.5	0	1	0	1	1	0.5	0	0	0	0	13	46.43%
23	Chen et al.	0.5	0.5	1	0	0	0.5	1	0	0	0	1	1	1	1	0	0	0	0	0	1	0	1	1	0.5	0	1	0	0	12	42.86%
24	He et al.	0.5	1	1	0	1	1	0.5	0	0	0	1	1	1	1	1	0	0	0	0	1	0	0.5	1	0.5	0	0	0	0	13	46.43%
25	Qi et al.	1	1	1	0	0	1	0.5	0	0.5	0	1	1	1	1	0	0	0	0	0	0	0	0.5	1	0	0	0	1	0	11.5	41.07%
26	Jin et al.	1	1	1	0	1	1	0.5	0	0	0	1	1	1	1	0.5	0	0	0	0	1	0	0.5	1	0.5	0	0	1	0	14	50.00%
27	Zhang et al.	0.5	1	1	0	1	1	0.5	0	1	1	1	1	1	1	0	0.5	0.5	0.5	0	1	0	0.5	1	0.5	0	1	0	0	16.5	58.93%
28	Yin et al.	1	1	1	0	0.5	1	0.5	0	0.5	0	1	1	1	1	1	0	0	0	0	1	0	0.5	1	0	0	0	0	0	13	46.43%
29	Ren et al.	0.5	1	1	0	0.5	1	1	0	0.5	0	1	1	1	1	0	0	0	0	0	1	0	0.5	1	0.5	0	1	0	0	13.5	48.21%
30	Lu et al.	1	1	1	0	0.5	1	0.5	0	0	0	1	1	1	1	0	0	0	0	0	1	0	0.5	1	0.5	0	0	0	0	12	42.86%
31	Tao et al.	1	1	1	0	0.5	1	0.5	0	0.5	0	1	1	1	1	0	0	0	0	0	0	0	0.5	1	0	0	1	0	0	12	42.86%
32	Xu et al.	0.5	0.5	1	0	0	0	0.5	1	1	1	1	1	1	1	0	0.5	0.5	0	0	1	0	1	1	0.5	0	1	1	0	16	57.14%
33	Liu et al.	0.5	0.5	1	0	0	0	0.5	1	1	1	1	1	1	1	0	0.5	0.5	0	0	1	0	1	1	0.5	0	1	1	0	16	57.14%
34	Wang et al.	1	1	1	0	1	1	0.5	0	0	0	1	1	1	1	0	0	0	0	0	1	0	0.5	1	1	0	1	0	0	14	50.00%
35	Zhang et al.	0.5	1	1	0	0	0	0.5	1	1	0	1	1	1	1	0	0.5	0.5	0	0	1	0	0.5	1	1	0	1	0	0	14.5	51.79%
36	Chen et al.	1	1	1	0	1	1	0.5	0	1	0	1	1	1	1	0	0	0	0	0	1	0	0.5	1	1	0	1	0	0	15	53.57%
37	Yang et al.	0.5	1	1	0	0.5	0	0.5	1	1	1	1	1	1	1	1	1	0.5	0	0	1	0	1	1	1	0	1	0	0	18	64.29%
38	Yang et al.	1	1	1	0	0	1	1	0.5	0.5	0.5	1	1	1	1	1	0	0	0	0	1	0	1	1	1	0	0	0	1	16.5	58.93%
39	Ma et al.	1	0.5	1	0	0	0	0.5	1	1	1	1	1	1	1	1	1	0.5	0	0	1	0	1	1	1	0	1	0	0	17.5	62.50%
40	Fu et al.	1	1	1	0	0	1	0.5	0.5	1	0	1	1	1	1	1	0	0	1	1	1	0	1	1	1	0	1	0	0	18	64.29%
41	Xiong et al.	1	0.5	1	0	0	1	1	1	0.5	1	1	1	1	1	0	1	0.5	0	0	1	0	1	1	1	0	1	0	0	17.5	62.50%
42	Chen et al.	1	0.5	1	0	0	1	1	0.5	0.5	0.5	1	1	1	1	0	0.5	0.5	0	0	1	0	0.5	1	0.5	0	0	0	0	14	50.00%
43	Xi et al.	1	0.5	1	0	0	1	1	0	1	1	1	1	1	1	1	1	0.5	0	0	1	0	1	1	1	0	1	1	0	19	67.86%
44	Liu et al.	0.5	0.5	1	0	0	1	0.5	0	1	0	1	1	1	0.5	0	0	0	0	0	0	0	1	1	0	0	1	0	0	11	39.29%
45	Li et al.	0.5	0.5	1	0	0	1	0.5	0.5	0.5	0.5	1	1	1	1	1	1	1	0	0	1	0	1	1	1	0	1	0	0	17	60.71%

46	Wei et al.	0.5	0.5	1	0	1	1	0.5	0.5	1	0.5	1	1	1	1	1	1	1	0	0	1	0	1	1	1	0	1	1	0	19.5	69.64%
47	Li et al.	1	0.5	1	0	1	1	1	0	0.5	0.5	0	0.5	1	1	0	0	1	0	0	1	0	1	0	0.5	0	0	0	0	12.5	44.64%
48	Wang et al.	0.5	1	1	0	1	1	1	0.5	1	1	1	1	1	1	1	1	1	1	1	1	0	1	1	1	0	1	1	1	24	85.71%
49	Zhou et al.	1	1	1	0	0	0	1	1	1	1	1	1	1	1	0	1	1	0	0	1	0	1	0.5	1	0	1	0	0	17.5	62.50%
50	Zuo et al.	0.5	1	1	0	1	1	1	0	0	0	1	1	1	1	0	0	0	1	0	1	0	1	1	1	0	1	0	0	15.5	55.36%
51	Xu et al.	0.5	1	1	0	1	1	1	1	1	1	1	1	1	1	1	1	1	0	1	1	0	1	1	1	0	1	1	1	23.5	83.93%
52	Luo et al.	0.5	1	1	0	1	1	1	1	1	1	1	1	1	1	1	1	1	0	0	1	0	1	0.5	1	0	1	1	1	22	78.57%
53	Liu et al.	1	1	1	0	0.5	1	1	0.5	1	0.5	1	1	1	0.5	1	1	0.5	0	0	1	0	1	0.5	1	0	1	1	1	20	71.43%
54	Gu et al.	1	0.5	1	0	1	1	1	1	1	1	1	1	1	1	1	1	1	0	1	1	0	1	1	1	0	1	1	1	23.5	83.93%
55	Lu et al.	0.5	0.5	1	0	0.5	1	1	1	1	1	1	1	1	1	1	1	1	0	1	1	0	1	1	1	0	1	1	1	22.5	80.36%
56	Hui et al.	1	0.5	1	0	0	0.5	1	0	0	0	1	0.5	0.5	0.5	0	0	0	0	1	0	0	0.5	0.5	0	0	0	0	0	8.5	30.36%
57	Chai et al.	1	0.5	1	0	0.5	0.5	1	0	0	0	1	1	0.5	1	1	1	1	0	0	1	0	0.5	0.5	1	0	1	0	0	15	53.57%
58	Li et al.	1	1	1	0	1	1	1	0	0	0	1	1	0.5	1	1	0	0	0	0	1	1	0.5	0.5	1	1	0	0	0	15.5	55.36%
59	Si et al.	1	1	1	0	1	1	1	0	0	0	1	1	1	1	1	0	0	0	0	1	1	0.5	0.5	1	1	1	0	0	17	60.71%
60	Wang et al.	0.5	1	1	0	1	1	0.5	0.5	0	0	1	1	1	1	0	0	0	0	0	1	0	1	1	1	0	1	0	0	14.5	51.79%
61	Xie et al.	1	1	1	0	1	1	1	0	0	0	1	1	1	1	0	0	0	0	0	0	0	1	1	0	0	1	0	0	13	46.43%
62	Chen et al.	0.5	1	1	0	1	1	1	1	0	0	1	1	1	1	0	0	0	0	0	1	0	1	1	1	0	1	0	0	15.5	55.36%
63	Chen et al.	0.5	1	1	0	1	1	1	0	0	0	1	1	1	0.5	0	0	0	0	0	1	0	1	1	1	0.5	1	0	0	14.5	51.79%
64	Zhan et al.	1	1	1	0	1	1	1	0	0	1	1	1	1	0.5	0.5	0	0	0	0	1	0	0.5	0.5	1	0	0	0	0	14	50.00%
65	Liu et al.	0.5	0.5	1	0	1	1	0.5	0	0	0	1	1	1	1	0.5	0	0	0	0	1	0	0.5	0.5	0.5	0	0	0	0	11.5	41.07%
66	Yan et al.	0.5	0.5	1	0	1	1	1	0	0	0	1	1	1	1	0.5	0	0	0	0	1	0.5	0.5	0.5	0.5	0.5	0	0	0	13	46.43%
67	Guan et al.	0.5	1	1	1	1	1	1	0.5	1	0.5	1	1	1	1	1	1	1	0	0	1	0	1	1	1	0	1	1	1	22.5	80.36%
68	Jiang et al.	0.5	1	1	0	1	1	1	1	1	1	1	1	1	1	1	1	1	0	0	1	0	1	1	1	0	1	1	1	22.5	80.36%
69	Gao et al.	1	1	1	0	0.5	1	1	1	1	1	1	1	1	1	1	1	1	0	0	1	0	1	1	1	0	1	1	1	22.5	80.36%
70	Yang et al.	1	1	1	0	1	1	1	0.5	1	0.5	1	1	1	1	1	1	1	0	0	1	0	0.5	0.5	1	0	1	0	0	19	67.86%

71	Hou et al.	1	1	1	0	1	1	1	0.5	0	1	1	1	1	1	1	0	0	0	0	1	0	1	1	1	0	1	1	1	19.5	69.64%
72	Cheng et al.	1	1	1	0	1	1	1	0.5	0	1	1	1	1	1	1	0	0	0	0	1	0	1	1	1	0	1	1	1	19.5	69.64%
73	Rui et al.	1	1	1	0	1	1	1	0.5	1	0	1	1	1	1	1	1	1	0	0	1	0	1	1	1	0	1	1	1	21.5	76.79%
74	Wang et al.	1	1	1	0	1	1	1	0.5	1	0.5	1	1	1	1	1	1	0	0	0	1	1	1	1	1	0	1	0	1	21	75.00%
75	Zhou et al.	1	1	1	0	1	1	1	0.5	1	1	1	1	1	1	1	1	0	0	0	1	0	1	1	1	0	1	1	1	21.5	76.79%
76	Wang et al.	1	1	1	0	0	1	1	0	0	0	1	1	1	1	1	0.5	0	0	0	1	0	1	0.5	1	0	1	0	0	15	53.57%
77	Tian et al.	1	1	1	0	1	1	1	0.5	0	0	1	1	1	1	0.5	0	0	0	0	1	0	1	1	0.5	0	0	0	0	14.5	51.79%
78	Zhou et al.	0.5	1	1	0	1	1	0.5	0.5	1	0	1	1	1	1	1	0	0	0	1	1	0.5	1	1	1	0	0.5	0	0	17.5	62.50%
79	Qi et al.	1	1	1	0	1	0	1	0	0.5	0	1	1	1	1	0	0	1	0	1	0	0	0.5	1	0	0	0.5	0	0	13.5	48.21%
80	Tang et al.	1	1	1	0	1	1	1	0.5	0.5	1	1	1	1	0.5	1	0.5	0.5	0	0	1	0	0.5	0.5	1	0	1	0	0	17.5	62.50%
81	Zhang et al.	1	1	1	0	1	1	0.5	1	0	0	1	1	1	1	0	1	1	0	0	1	0	1	1	0	0	1	0	0	16.5	58.93%
82	Yang et al.	1	1	1	0	0	0	1	1	0	0	1	1	1	1	1	1	1	0	0	1	0	0.5	1	0	0	1	0	0	15.5	55.36%
83	Xuan et al.	1	1	1	0	1	1	1	0.5	0.5	0.5	1	1	1	1	1	1	0	0	0	1	0	1	1	1	0	1	1	1	20.5	73.21%
84	Wang et al.	1	0.5	1	0	1	1	1	0.5	0.5	0	1	1	1	1	1	1	0	0	0	1	0	1	1	1	0	1	1	1	19.5	69.64%
85	Zhou et al.	1	1	1	0	1	1	1	0.5	0.5	0	1	1	1	1	1	1	0	0	0	1	0	1	0	0	0	1	1	1	18	64.29%
86	Wang et al.	1	1	1	0	0	1	1	0.5	0.5	0	1	1	1	1	1	0	1	0	0	1	0	1	0	0	0	1	1	1	17	60.71%
87	Zhang et al.	1	1	1	0.5	0	1	1	0.5	0	0	1	1	1	0	1	1	1	0	0	0	0	1	0	0	0	1	1	1	16	57.14%
88	Zhou et al.	1	1	1	0	0	1	1	0.5	0	0	1	1	1	1	1	1	1	0	0	0	0	1	0	0	0	1	1	1	16.5	58.93%
89	Liu et al.	1	1	1	0.5	0	1	1	1	0.5	1	1	1	1	1	1	1	1	0	0	1	1	1	0.5	1	0	1	1	1	22.5	80.36%
90	Chen et al.	1	1	1	0	1	1	1	0.5	1	0.5	1	1	1	1	1	1	0	0	0	1	0	0.5	1	1	0	1	0	0	18.5	66.07%
91	Tian et al.	1	1	1	0	1	1	1	0.5	0.5	0.5	1	1	1	1	1	1	0	1	0	1	0	1	0	1	0	1	1	1	20.5	73.21%
92	Zeng et al.	1	1	1	0	1	1	1	0	0.5	0	1	1	1	1	0	1	1	0	0	1	0	1	0.5	0	0	1	1	1	18	64.29%
93	Cui et al.	1	1	1	0	0	0	0	0.5	0.5	0.5	1	1	1	1	1	1	0	0	0	1	0	1	0.5	1	0	1	1	1	17	60.71%
94	Sun et al.	1	0.5	1	0	1	1	1	0.5	0.5	0	1	1	1	1	1	0.5	1	0	0	1	0	1	1	1	0	1	1	1	20	71.43%
95	Bao et al.	1	1	1	0	0	1	1	1	0.5	1	1	1	1	1	1	1	0	0	0	1	0	1	1	1	0	1	1	1	20.5	73.21%

96	Jiang et al.	1	1	1	0	1	1	1	0.5	0.5	0.5	1	1	1	1	1	1	0.5	1	0	1	0	1	1	1	0	1	1	1	22	78.57%
97	Jia et al.	1	1	1	0	1	1	1	0.5	1	0.5	1	1	1	1	1	1	1	0	0	1	0	1	1	1	0	1	0	0	20	71.43%
98	Mu et al.	1	1	1	0	0	0	1	1	0	0	1	1	1	1	1	1	1	0	0	1	0	1	1	1	0	1	0	0	17	60.71%
99	Liu et al.	1	1	1	0	0	0	1	0	0	0	1	1	1	1	1	1	1	0	0	1	0	1	1	0	0	1	1	0	16	57.14%
100	Shi et al.	1	1	1	0	1	1	1	0	0	0	1	1	1	1	0	1	1	0	0	1	0	1	0	0	0	1	0	1	16	57.14%
101	Wang et al.	0.5	0.5	1	0	0	1	1	0.5	1	0	1	0.5	0.5	1	1	0.5	0	0	0	0	0	0.5	1	0	0	0	0	0	11.5	41.07%
102	Liu et al.	0.5	0.5	1	0	0	1	1	0.5	1	1	1	0.5	0.5	1	0.5	0	0	0	0	1	0	1	0	1	0	1	1	0	15	53.57%
103	Shi et al.	0.5	0.5	1	0	0	1	1	0.5	1	1	1	1	1	0.5	0	0.5	0.5	0	0	1	0	1	1	1	0	1	1	0	17	60.71%
104	Wu et al.	0.5	0.5	1	0	0	1	0.5	0.5	0.5	1	1	1	1	1	0	1	0.5	0	0	1	0	1	1	1	0	1	1	0	17	60.71%
105	Yang et al.	1	0.5	1	0	1	1	0.5	0.5	0.5	1	1	1	1	0.5	1	0	0	0	0	1	0	1	1	0.5	0	1	1	0	17	60.71%
106	Gong et al.	1	0.5	1	0	1	1	1	0.5	1	1	1	1	1	1	1	1	1	0	0	1	0	1	1	1	0	1	0	0	20	71.43%
107	Pang et al.	0.5	0.5	1	0	1	1	0.5	0	0	0	1	0.5	0.5	0	0	0	0	1	1	1	0	0.5	1	0.5	0	1	1	0	13.5	48.21%
108	Peng et al.	1	1	1	0	1	1	1	0	0	0	1	1	0.5	0	0	0	0	0	0	1	0	1	1	0.5	0	0.5	1	1	14.5	51.79%
109	Pei et al.	0.5	0.5	1	0	1	1	0.5	0	0	0	1	0.5	0.5	0.5	0	0	0	0	0	1	0	0.5	1	0.5	0	0	1	0	11	39.29%
110	Zhang et al.	0.5	1	1	0	0.5	1	1	0.5	1	1	1	0.5	0.5	0.5	0	1	0.5	0	0	1	0	1	1	1	0	1	1	0	17.5	62.50%
111	Qi et al.	0.5	1	1	0	0	1	0.5	0.5	1	1	1	1	1	1	1	1	1	1	1	1	0	1	1	1	1	1	1	0	22.5	80.36%
112	Li et al.	0.5	1	1	0	0	1	0.5	0.5	0.5	1	1	1	0.5	1	1	0.5	1	0	0	1	0	1	1	1	0	1	0	0	17	60.71%
113	Gao et al.	0.5	0.5	1	0	0	1	1	0.5	0.5	1	1	1	1	1	1	0	0	0	0	1	0	0.5	1	0.5	0	1	0	0	15	53.57%
114	Dai et al.	1	1	1	0	1	1	1	0.5	0.5	1	1	1	1	1	0	1	0.5	0	0.5	1	0	1	1	0.5	0	0.5	0	1	19	67.86%
115	Fei et al.	1	1	1	0	1	1	0.5	0.5	0.5	1	1	1	1	1	1	0.5	0.5	0	1	1	0	1	1	0.5	0	0.5	1	1	20.5	73.21%
116	Gong et al.	1	1	1	0	1	1	1	0.5	0.5	1	1	1	1	1	1	0.5	0.5	0	1	1	0	1	1	0.5	0	1	1	1	21.5	76.79%
117	Hu et al.	1	1	1	0	1	1	1	0.5	0.5	1	1	1	1	1	1	0.5	0.5	0	1	1	0	1	1	0.5	0	0.5	1	1	21	75.00%
118	Hu et al.	1	1	1	0	1	1	1	0.5	0.5	1	1	1	1	1	1	1	1	0.5	1	1	0	1	1	1	0	0.5	0	1	22	78.57%
119	Huang et al.	1	0.5	1	0	1	1	1	0.5	0.5	0	1	1	1	1	1	1	0.5	0	1	1	0	1	1	1	0	1	1	0	20	71.43%
120	Huo et al.	1	1	1	0	1	1	1	0.5	1	0.5	1	1	1	1	1	1	0.5	1	1	1	0.5	1	1	1	0	1	0	1	23	82.14%

121	Kong et al.	1	1	1	0	1	1	1	0.5	0.5	0.5	1	1	1	1	1	1	0.5	0	1	1	0	1	1	1	0	1	1	1	22	78.57%
122	Li et al.	1	1	1	0	1	1	1	0.5	1	1	1	1	1	1	1	1	0.5	0	1	1	0	1	1	1	0	1	1	1	23	82.14%
123	Chen et al.	1	1	1	0	1	0	1	0.5	1	1	1	1	1	1	1	0.5	0.5	0	1	0	0	1	1	0	0	0.5	0	1	18	64.29%
124	Li et al.	1	1	1	0	0	0.5	1	1	1	0.5	1	1	1	1	1	1	1	0	0	1	0	1	1	1	0	1	1	1	21	75.00%
125	Li et al.	1	1	1	0	0	0.5	1	1	1	0.5	1	1	1	1	1	1	1	0	0	1	0	1	1	1	0	1	1	1	21	75.00%
126	Liang et al.	1	1	1	0	0.5	0.5	1	1	1	1	1	1	1	1	1	1	1	1	1	1	0	1	1	1	0	1	1	1	24	85.71%
127	Lin et al.	1	1	1	0	0	0.5	1	1	1	1	1	1	1	0.5	1	1	1	1	1	1	0	1	0.5	1	0	1	0	1	21.5	76.79%
128	Liu et al.	1	1	1	0	0.5	0.5	1	1	1	1	1	1	1	1	1	1	1	0.5	0.5	1	0	0.5	1	1	0	1	1	1	22.5	80.36%
129	Liu et al.	1	1	1	0	0	0.5	1	1	1	1	1	1	1	1	1	1	1	1	0.5	1	0	1	1	1	0	1	0	1	22	78.57%
130	Liu et al.	1	1	1	0	0	0.5	1	1	1	1	1	1	1	1	1	1	1	1	0.5	1	0	1	1	1	0	1	0	1	22	78.57%
131	Nie et al.	1	1	1	0	0	0.5	1	1	1	1	1	1	1	1	1	1	1	0	0	1	0	1	1	1	0	1	0	1	20.5	73.21%
132	Qiu et al.	1	1	1	0	0	0.5	1	1	1	1	1	1	1	1	1	1	1	0.5	1	1	1	1	1	1	0	1	0	1	23	82.14%
133	Shang et al.	1	1	1	0	1	1	1	1	1	1	1	1	1	1	0.5	1	1	1	1	1	1	1	1	1	0	1	1	1	25.5	91.07%
134	Shao et al.	1	1	1	0	1	1	1	1	1	1	1	1	1	1	1	1	1	1	1	1	1	1	1	1	0	1	1	1	26	92.86%
135	Shi et al.	1	0.5	1	0	1	0	1	0.5	0.5	0	1	1	0.5	1	0	1	0	0	0	1	0	1	1	0	0	1	1	1	16	57.14%
136	Shi et al.	1	1	1	0	1	0	1	0.5	1	0.5	1	1	0.5	1	0.5	0.5	0	0	0	1	0	1	1	1	0	1	1	1	18.5	66.07%
137	Shu et al.	1	1	1	0	1	0	1	0.5	0.5	0.5	1	0.5	1	1	1	1	0.5	1	0.5	1	0	1	1	0.5	0	1	1	1	20.5	73.21%
138	Shu et al.	1	1	1	0	1	0	1	0.5	1	0.5	1	1	1	1	1	1	0.5	1	0.5	1	0	1	1	1	0	1	1	1	22	78.57%
139	Wang et al.	1	0.5	0.5	0	0.5	0	1	0.5	0.5	0.5	1	0.5	1	1	1	0.5	0.5	0	0	1	0	0.5	1	1	0	1	1	1	17	60.71%
140	Wu et al..	1	0.5	0.5	0	0.5	0	0.5	0.5	1	0.5	1	1	0.5	1	0.5	1	0.5	0	0	1	0	0.5	1	0.5	0	1	1	1	16.5	58.93%
141	Xu et al.	1	0.5	0.5	0	1	0	0.5	0.5	0.5	0.5	1	1	0.5	1	0.5	0.5	0	0	0	1	0	0.5	0.5	1	0	1	0	0	13.5	48.21%
142	Ye et al.	1	0.5	0.5	0	0.5	0	1	0.5	1	0.5	1	1	1	1	0.5	0.5	0	0	0	1	0	0.5	1	1	0	1	1	1	17	60.71%
143	Lang et al.	1	1	1	0	1	0	0.5	0.5	0.5	0.5	1	0.5	1	1	1	1	0	0	0	1	0	1	0.5	1	0	1	0	0	16	57.14%
144	Qi et al.	1	1	1	0	0.5	0	0.5	0.5	1	0.5	1	1	0.5	1	0.5	1	0	0	0	1	0	0.5	0.5	1	0	1	0	0	15	53.57%
145	Rao et al.	1	1	1	0	0.5	0	0.5	0.5	0.5	0.5	1	1	0.5	1	0	0.5	0	0	0	0	0	0.5	1	1	0	1	0	0	13	46.43%

146	Ye et al.	0.5	1	1	0	0.5	0.5	0.5	0.5	1	0.5	1	1	0.5	1	1	0.5	0	1	0.5	1	0	1	1	0.5	0	1	1	1	19	67.86%
147	Zeng et al.	0.5	1	1	0	0.5	0	0	0.5	1	0.5	1	0.5	0.5	0.5	1	0.5	0	0.5	0.5	0	0	1	1	0	0	1	1	1	15	53.57%
148	Zhang et al.	1	1	1	0	1	0.5	0.5	0.5	1	0.5	1	1	0.5	1	1	0.5	0	1	0.5	1	0	1	1	0.5	0	1	1	1	20	71.43%
149	Zhang et al.	1	1	1	0	0.5	0.5	0.5	0.5	0.5	0.5	1	1	0.5	1	0	0.5	0	0.5	0.5	1	0	1	1	0.5	0	1	1	1	17.5	62.50%
150	Zhao et al.	0.5	1	1	0	0.5	0.5	0.5	0.5	0.5	0.5	1	1	0.5	1	0	0.5	0	0	0.5	1	0	1	1	0.5	0	1	1	1	16.5	58.93%
151	Zhou et al.	1	1	1	0	0.5	1	0.5	0.5	0.5	0.5	1	1	0.5	1	1	0.5	0	0	0.5	1	0	1	1	0.5	0	1	1	1	18.5	66.07%
152	Zhu et al.	1	1	1	0	0.5	1	0.5	0.5	0.5	0.5	1	1	0.5	1	0	0.5	0	0	0.5	1	0	1	1	0.5	0	1	1	1	17.5	62.50%
153	Zhu et al.	1	1	1	0	0.5	1	1	0.5	0.5	1	1	0.5	1	1	1	0.5	0	0	0.5	1	0	1	1	0.5	0	1	1	1	19.5	69.64%
154	Wang et al.	1	1	1	0	0	0	0	0.5	0.5	0.5	1	1	1	1	0.5	0.5	0	0	0	1	0	1	0.5	0.5	0	1	0	0	13.5	48.21%
