# Supplementary material for: A Systematic Review and Quality Assessment of Pharmacoeconomic Publications for China Compared to Internationally: Is the Quality of Evidence-Base Sufficient for Health Technology Assessment?
Source: Int J Health Policy Manag. 2025 Apr 28;14:8656. doi: 10.34172/ijhpm.8656 (PMC12257205; doi:10.34172/ijhpm.8656)
Supplement: Supplementary file 3 — describes the lists of studies included in the systematic review. [file ijhpm-14-8656-s003.pdf]

Article title: A Systematic Review and Quality Assessment of Pharmacoeconomic Publications for China Compared to Internationally: Is the Quality of Evidence-Base Sufficient for Health Technology Assessment?

Journal name: International Journal of Health Policy and Management

Authors' information: Zhixin Fan[1,2,3]¶, Xu Si[1,2,3]¶, Zhongxiang Wang[4], Liwei Zhang[1,2,3], Junyang Liu[1,2,3], Qing He[1,2,3], Matthew Franklin[5], Qiang Sun[1,2,3,6]*, Jia Yin[1,2,3]*

[1]Department of Social Medicine and Health Management, School of Public Health, Cheeloo College of Medicine, Shandong University, Jinan, China.

[2]NHC Key Lab of Health Economics and Policy Research, Shandong University, Jinan, China.

[3]Center for Health Management and Policy Research, Shandong University, Shandong Provincial Key New Think Tank, Jinan, China.

[4]Zhucheng Shiqiaozi Health Hospital, Zhucheng, China.

[5]Health Economics and Decision Science (HEDS), School of Health and Related Research (ScHARR), University of Sheffield, Sheffield, UK.

[6]China National Health Development Research Center, Beijing, China.

***Correspondence to:** Qiang Sun, qiangs@sdu.edu.cn & Jia Yin, yinjia@sdu.edu.cn

¶ Both authors contributed equally to this paper.

Citation: Fan Z, Si X, Wang Z, et al. A systematic review and quality assessment of pharmacoeconomic publications for China compared to internationally: Is the quality of evidence-base sufficient for health technology assessment? Int J Health Policy Manag. 2025;14:8656. doi:10.34172/ijhpm.8656

Supplementary file 3

1. Lists of studies included in systematic review.

1. Chen Y, Li H, Liu C, Zheng L. Pharmacoeconomic evaluation of Markov model of Addy injection adjuvant therapy for cervical cancer. China Health Care & Nutrition. 2018;28(4):1004-7484.
2. Yu T, Lu X, Shan L. Clinical efficacy and pharmacoeconomics of concomitant chemoradiotherapy with pemetrexed or etoposide plus cisplatin for locally advanced lung adenocarcinoma. Chinese General Practice. 2018;21(23):2871-2875.
3. Xie X, Dong Y, Pan X, Zhang F, Mu D. Cost-effectiveness analysis of mailuoning injection and compound danshen injection in the treatment of ischemic stroke. China Pharmacy. 2018;29(21):2962-2965.
4. Li H, Luo M, Xiao C, Juan D, Xu L, Li X, et al. Cost-effect analysis of vancomycin and linezolid in treatment of MRSA infection. China Journal of Modern Medicine. 2018;28(6):1005-8982.
5. Qin Z, Zhan M, He Z, Xu T. Cost-effectiveness analysis of SOX regimen versus capeOX regimen for metastatic colorectal cancer. China Pharmacy. 2018;29(06):779-783.
6. Chen C, Liu J, Zhang Y, Lin H. Economic evaluation of three treatment regimens for advanced non-small cell lung cancer. Journal of Basic Chinese Medicine. 2018;24(04):507-509+529.
7. Xie L, Yu L, Wang D, Zhang X. Cost-effectiveness analysis on nutrition therapy of perioperative patients with gastrointestinatumors receiving parenteral nutrition: compounded parenteral vs pre-mixed multi-chamber bag. Chinese Journal of Cancer Prevention and Treatment. 2018,25(24),1729-1732.
8. Xu S, Lyu S, Wang Y, Li D, Xu P. Analysis of pharmacoeconomic of three therapeutic schemes for mammary cancer. Chinese Journal of Modern Applied Pharmacy. 2018;35(02):271-274.

9. Wang C, Zhang J, Zhao Y, Li M, Jiang J. Efficacy, safety and pharmacoeconomics analysis of triple and quadruple therapies based on rabeprazole for treatment of helicobacter pylori infection with peptic ulcer. Chinese Journal of Clinical Research. 2018;31(10):1379-1382.
10. Hu Y, Shen Li, Shen J, Peng Y, Liu J. Cost-effectiveness analysis of three 5-HT3 receptor antagonists in preventing mFOLFOX6 chemotherapy-induced nausea and vomiting. Chinese Journal of Clinical Oncology and Rehabilitation. 2018;25(01):33-35.
11. Tian Y, Cui X, Liu L, Zhang C. Cost-effectiveness analysis of the six kinds of hepatoprotective drugs in the treatment of drug-induced liver injury in China based on the decision tree model. Chinese Journal of Drug Application and Monitoring. 2018;15(03):131-135.
12. Yuan Y, Liu M, Wang X, Zeng R. Pharmacoeconomic evaluation of hepatoprotective Chinese medicine combined with nucleoside analogues in the treatment of chronic hepatitis B. China Health Care & Nutrition. 2019;29(29):1004-7484.
13. Xu L, Wang X, Wei J, Wang W, Zhou J, Lu S, et al. A Meta-analysis-based pharmacoeconomic evaluation of simotang oral liquid in the treatment of gastrointestinal functional diseases and jaundice in children. China Journal of Pharmaceutical Economics. 2022;17(09):40-48.
14. Wei C, Luo Q, Hu M, Liang W, Zhou N. Pharmacoeconomic evaluation of shenshuaining capsule in the treatment of chronic renal failure based on Meta-analysis. Chinese Pharmaceutical Affairs. 2022;36(08):852-861.
15. Chen Z, Jiang X, He X. Meta-analysis and pharmaceutical economic evaluation of clotrimazole vaginal swelling suppository and clotrimazole vaginal tablets in the treatment of candidal vaginosis. Journal of China Prescription drug. 2022,20(08):41-44.
16. Lu Y, Lu W, Wang Y, Jiang F. Net Meta-analysis and pharmacoeconomic evaluation of kuhuang granules for jaundice viral hepatitis. China Journal of Pharmaceutical Economics. 2022;17(08):5-10.
17. Jing J, Sun L. Network Meta analysis and pharmacoeconomic evaluation of three drugs in the treatment of acute ischemic stroke. Chinese Journal of New Drugs.

2022;31(14):1441-1448.

18. Wang Z, Li Y, Pang S. Meta analysis and pharmacoeconomic evaluation of azithromycin combined with oral Chinese patent medicine in the treatment of mycoplasma pneumonia in children. China Journal of Pharmaceutical Economics. 2022;17(07):25-30+38.
19. Ma L, Wang M, Sun X, Li M, Zhang X, Zhao H, et al. Pharmacoeconomics evaluation of shensong yangxin capsule in the treatment of ventricular premature beat based on Meta-analysis. Chinese Journal of Integrative Medicine on Cardio-Cerebrovascular Disease. 2022;20(08):1352-1358.
20. Zhang Z, Chen Z. Meta-analysis and evidence-based pharmacoeconomics study of compound calcium carbonateparticles and calcium carbonate D-3 particles in the treatment of infantile rickets. China Journal of Pharmaceutical Economics. 2021;16(09):20-27+40.
21. Jing J, Hu X, Shi J, Sun H, Cui Y, Li X, et al. Reticular Meta-analysis and pharmacoeconomic evaluation of three drugs to improve blood circulation combined with basic therapy for acute cerebral infarction. Drug Evaluation Research. 2021;44(03):617-627.
22. Zhang A, Wang J, Li C, Liu Z, Zhou X, Lu W. Meta-analysis and pharmacoeconomic evaluation of ilaprazole in the treatment of duodenal ulcer. China Journal of Pharmaceutical Economics. 2020;15(05):24-29+45.
23. Chen P, Chen F, Zhou B. Pharmacoeconomic evaluation of ligustrazine hydrochloride injection in the treatment of cardiovascular diseases based on Meta-analysis. Chinese Journal of Pharmacoepidemiology. 2018;27(05):298-304.
24. He X, Xie L. Cost-effectiveness analysis of four premixed insulins in patients with type 2 diabetes. Journal of China Prescription Drug. 2020;18(11):59-60.
25. Qi M, Li M, Yang F, Liu S, Lu Q, Tao J. Clinical effect of albumin-bound paclitaxel combined with cisplatin and apatinib in the treatment of advanced cervical cancer. Chinese Journal of Modern Applied Pharmacy. 2020;37(17):2143-2147.
26. Jin J, Shen J, Gu B, Wang H. Cost-effectiveness analysis of medium/long-chain propofol combined with sufentanil for sedation and analgesia in patients with

craniocerebral injury. China Pharmacist. 2020;23(06):1111-1114.

27. Zhang J, Ren L, Liu D, Liu Z, Zhang X, Wu Y, et al. Cost-effectiveness analysis of anlotinib in treating advanced non-small cell lung cancer. Chinese Journal of New Drugs and Clinical. 2020;39(06):380-384.
28. Yin P, Jiang Z, Mo Y. Pharmacoeconomic analysis of long-acting anticholinergic drugs/long-acting β2 receptor agonists bronchodilator in treatment of chronic obstructive pulmonary disease in group d. China Journal of Pharmaceutical Economics.2020;15(09):27-29+35.
29. Ren F, Wang C, Tan X, Wang H. Pharmacoeconomic evaluation of magnesium lsoglycyrrhizinate preventing liver damage induced by chemotherapeutic drugs for gastric cancer. China Pharmacy. 2020;31(13):1613-1617.
30. Lu J. Clinical effect and cost-effectiveness analysis of domestic and imported esomeprazole in the treatment of upper gastrointestinal bleeding. China Journal of Pharmaceutical Economics. 2021;16(12):1673-5846.
31. Tao Z, Chen J, Li X, Mo C, Su C, Huang X. Clinical efficacy of dexamethasone combined with ctx in the treatment of middle-aged and elderly type 2 diabetic patients with idiopathic membranous nephropathy. Chinese General Practice. 2021;24(17):2169-2173.
32. Xu W, Dai W, Han B, Guo M, Cui X. Cost-effectiveness analysis of four immunosuppressive agents in treatment of idiopathic membranous nephropathy.herald of medicine. 2021,40(10);1418-1422.
33. Liu S, Cui X, Zhang Z, Xu W, Guo X, Li J. Cost-effectiveness analysis of three drugs for Clostridium difficile infection based on decision tree model. Chinese Journal of Drug Application and Monitoring. 2021;18(06):349-354.
34. Wang X, Yang Y, Wu R, Sun J, Xu Y. Pharmacoeconomics study of sofren injection and danhong injection in the treatment of stable angina pectoris of coronary heart disease. Evaluation and Analysis of Drug-Use in Hospitals of China. 2021;21(10):1223-1226.

35. Zhang R, Wu B. Clinical efficacy and economic analysis of three ovulation induction drugs in ivf-et/icsi--a retrospective analysis based on real world. Chinese Journal of Modern Applied Pharmacy. 2021;38(17):2128-2133.

36. Cheng S，Deng S，Yin T. Cost effectiveness analysis of domestic and importedtenofovir disoproxil fumarate for chronic hepatitis B. Chinese Journal of Hospital Pharmacy. 2021;41(10):1040-1043+1063.

37. Yang F, He X, Wu J. Economic evaluation of novel direct-acting antivirals in the treatment of chronic hepatitis c virus genotype 1b in china based on Markov model. Chinese Pharmaceutical Journal. 2019;54(15):1276-1284.

38. Yang L, Zhang L, Yang L. To compare the cost-effectiveness of PTX and cinacalcet for patients with hemodialysis complicated with secondary hyperparathyroidism--a study based on real world data. Chinese Journal of Endocrine Surgery. 2021;(6):661-665.

39. Ma A, Zhang J. Pharmacoeconomic evaluation of compacept and rezumumab in the treatment of age-related macular degeneration. China Journal of Pharmaceutical Economics. 2018;13(09):27-31.

40. Fu L, Chen P, Li H, Zhang J, Xiong X, Ma A. Economic evaluation of the combination of dezocine and sufentanil vs sufentanil alone for postoperative analgesia. Chinese Journal of New Drugs. 2019;28(01):121-125.

41. Xiong X, Li H, Ma A. The economical evaluation of dutasteride versus finasteride in patients with benign prostatic hypertrophy. Chinese Journal of New Drugs. 2019;28(07):891-896.

42. Chen P, Ma A. Cost effectiveness analysis of elbasvir/grazoprevir for the treatment of chronic hepatitis C. Chinese Journal of New Drugs. 2020;29(04):470-476.

43. Xi X, Xie W, Liu Y, Huang R, Chen L. Pharmacoeconomic evaluation of rosiglitazone sodium and metformin in the treatment of type 2 diabetes mellitus. China Pharmacy. 2020;31(02):212-216.

44. Tan B，Ren M，Xuan J. Economic evaluation of hemocoagulase bothrops atrox for injection for surgery application based on the real world. China Journal of Pharmaceutical Economics. 2020;15(03):10-14+39.

45. Li X, Xiao F, Li M, Li R, Han X, Zhao K. The economic evaluation of targeted drugs for chronic myeloid leukemia. China Journal of Pharmaceutical Economics. 2021;16(05):5-11.

46. Wei X, Yang L. Cost-utility analysis of the pan-genotypic direct-acting antivirals in the treatment of chronic hepatitis c infection. China Pharmacy. 2021;32(08):979-985.

47. Li W, Meng Z, Liu Z. Pharmacoeconomic analysis of rivaroxaban and warfarin for stroke prevention and treatment in patients with atrial fibrillation. China Journal of Pharmaceutical Economics. 2021;16(02):28-31.

48. Li W, Meng Z, Liu Z. Pharmacoeconomic analysis of rivaroxaban and warfarin for stroke prevention and treatment in patients with atrial fibrillation. China Journal of Pharmaceutical Economics. 2021;16(02):28-31.

49. Zhou X, Sun G. Cost-utility analysis of trametinib plus dabrafenib versus vemurafenib in the treatment of metastatic melanoma with BRAF V600 mutation. Chinese Journal of Hospital Pharmacy. 2022,42(17):1830-1833.

50. Zuo K, Zhang C, Zhang M, Zhang H, Wang Z, Pan J, et al. Pharmacoeconomic evaluation of liuwei dihuang pills combined with hypoglycemic agents in the treatment of type 2 diabetes mellitus. China Pharmacist. 2020;23(09):1798-1804.

50. Xu X, Bao Y, Xu K, et al. Economic Value of Fosaprepitant-Containing Regimen in the Prevention of Chemotherapy-Induced Nausea and Vomiting in China: Cost-Effectiveness and Budget Impact Analysis. Front Public Health. 2022;10:913129.

51. Luo S, Weng X, Lin S, et al. Evaluation of osimertinib for advanced non-small cell lung cancer with leptomeningeal metastases: a cost-effectiveness and budget

impact analysis. Int J Clin Pharm. 2022;44(1):192-200.

52. Liu K, Zhu Y, Zhu H. Immunotherapy or targeted therapy as the first-line strategies for unresectable hepatocellular carcinoma: A network meta-analysis and cost-effectiveness analysis. Front Immunol. 2023;13:1103055. Published 2023 Jan 11.

53. Gu YL, Sun ZX, Sun Y, et al. A real-world cost-effectiveness analysis of nebulized budesonide and intravenous methylprednisolone in acute exacerbation of chronic obstructive pulmonary disease. Front Pharmacol. 2022;13:892526.

54. Lu L, Li Y, Jin Q, et al. Safflor yellow treating angina pectoris: A pharmacoeconomic evaluation and network meta-analysis. Medicine (Baltimore). 2022;101(41):e31036.

55. Hui Q, Song J, Yang Y, et al. Pharmaceutical characteristics and pharmacoeconomics comparison of 6 commonly used 5-HT3 receptor antagonists. Chinese Journal of Medicinal Guide. 2019;21(11):687-691.

56. Chai Q, Du J, Zhang C, et al. Cost-effectiveness analysis of oral iron supplements of iron-deficiency anemia in pregnancy based on decision tree. Chinese Journal of New Drugs. 2019;28(19):2428-2432.

57. Li W, Zhang Y, Bu S, et al. Minimum cost analysis of 3 ppis in the prevention of stress ulcer in cervical spinal cord injury patients. China Pharmacist. 2019;22(05):870-872.

58. Si R, Yang H, Wu R, et al. Analysis on clinical effects and pharmacoeconomics of late non-small cell lung cancer chemotherapy and chemotherapy combined with gefitinib. China Journal of Pharmaceutical Economics. 2019;14(05):45-49.

59. Wang W, Yu J, Wang P, et al. Cost-effectiveness analysis of different lipid-lowering regimens in patients with acute coronary syndrome. China Journal of Pharmaceutical Economics. 2019;14(08):26-29+57.

60. Xie Y, Ye D, Zhang H, et al. Study on the pharmacoeconomic analysis of domestic and imported pemetrexed in advanced non-small cell therapy. Chinese Journal of Hospital Pharmacy.2019;39(03):278-281.
61. Chen S, Dai L, Li D, et al. Cost-minimization analysis of interferon-α1b combined with oseltamivir in treating pneumonia caused by influenza virus in children. Chinese Journal of Modern Applied Pharmacy. 2019;36(08):970-973.
62. Chen Z, Zhang W, Peng W. Pharmacoeconomic study on different cephalosporins in preventing cesarean section infection. Evaluation and Analynisis of Drug-Use in Hospitals of China. 2020;20(01):34-36+39.
63. Zhan S, Li H, He A, et al. Pharmacoeconomic study on the treatment of herpes zoster with different quality levels of famciclovir, aciclovir and valaciclovir. Chinese Journal of Modern Drug Application. 2020;14(12):217-219.
64. Liu Z, Zhu S, He X. Cost-effectiveness analysis of three medication regimens for essential hypertension in our hospital in 2017. Chinese Journal of Rural Medicine and Pharmacy. 2020;27(09):40-41.
65. Yan F, Xu W, Le L. Effect evaluation and cost-effectiveness analysis on intervention of rational drug use by pharmacists in perioperative period of cesarean section. China Modern Medicine. 2020;27(22):166-169.
67. Guan H, Liu G, Xie F, et al. Cost-effectiveness of Osimertinib as a Second-line Treatment in Patients With EGFR-mutated Advanced Non-Small Cell Lung Cancer in China. Clin Ther. 2019;41(11):2308-2320.e11.
68. Jiang Y, Cai D, Chen D, et al. Economic evaluation of remdesivir for the treatment of severe COVID-19 patients in China under different scenarios. Br J Clin Pharmacol. 2021;87(11):4386-4396.
69. Gao T, Liu J, Wu J. Cost-Effectiveness Analysis of Dabrafenib Plus Trametinib and Vemurafenib as First-Line Treatment in Patients with BRAF V600

Mutation-Positive Unresectable or Metastatic Melanoma in China. Int J Environ Res Public Health. 2021;18(12):6194.

70. Yang L, Wu J. Cost-effectiveness of rivaroxaban compared with enoxaparin plus warfarin for the treatment of hospitalised acute deep vein thrombosis in China. BMJ Open. 2020;10(7):e038433.

71. Hou X, Wan X, Wu B. Cost-Effectiveness of Canagliflozin Versus Dapagliflozin Added to Metformin in Patients With Type 2 Diabetes in China. Front Pharmacol. 2019;10:480.

72. Cheng H, Wan X, Ma J, et al. Cost-effectiveness of Insulin Degludec Versus Insulin Glargine in Insulin-naive Chinese Patients With Type 2 Diabetes. Clin Ther. 2019;41(3):445-455.e4.

73. Rui M, Wang Y, Fei Z, et al. Economic Evaluation of Rituximab + Recombinant Human Thrombopoietin vs. Rituximab for the Treatment of Second-Line Idiopathic Thrombocytopenic Purpura in China. Front Med (Lausanne). 2021;8:657539.

74. Wang Y, Rui M, Yang L, et al. Economic Evaluation of First-Line Atezolizumab for Extensive-Stage Small-Cell Lung Cancer in the US. Front Public Health. 2021;9:650392.

75. Zhou T, Wang X, Cao Y, et al. Cost-effectiveness analysis of sintilimab plus bevacizumab biosimilar compared with lenvatinib as the first-line treatment of unresectable or metastatic hepatocellular carcinoma. BMC Health Serv Res. 2022;22(1):1367.

76. Wang Y , Chen B , Zhao M, et al. Pharmacoeconomic evaluation of rituximab (hanlikang) for patients with rheumatoid arthritis. Asian Journal of Social Pharmacy. 2020;15(1):8.

77. Tian Y, Fang Y, Hu M. Cost-effectiveness analysis of danshen ligustrazine injection and shenxiong glucose injection for cerebral infarction. China Pharmacy. 2018;29(04),487-49.53(08):652-658.

78. Zhou J, Liu F, Jiang W, et al. Cost-effectiveness analysis of jingshu granules in the treatment of cervical radiculopathy basedon randomized clinical trials. China Journal of Pharmaceutical Economics. 2018;13(11):50-54.

79. Qi Y, Gao W, Li H, et al. Efficacy and economic evaluation of tramadol and hydromorphone combined with flurbiprofen axetil in the treatment of pcia for postoperative cesarean. Chinese Pharmaceutical Journal. 2018;38(07):652-658.

80. Tang S, Chen W. Pharmacoeconomic study on 3 chemotherapy regimens for advanced pancreatic cancer in china based on Markov model. China Pharmacy. 2018;29(06):784-789.

81. Zhang K, Cao S, Zhang J, et al. Analysis of decision tree model for drug treatment of mycoplasma pneumonia in children. Hospital Administration Journal of Chinese People's Liberation Army. 2018;25(05):401-405.

82. Yang A, Chen L, Liang N, et al. Cost-effectiveness analysis of compound xueshuantong capsule in the treatment of diabetic retinopathy. Chinese Journal of Hospital Pharmacy. 2018;38(07):763-768.

83. Xuan J, Huang M, Lu Y, et al. Economic Evaluation of Safflower Yellow Injection for the Treatment of Patients with Stable Angina Pectoris in China: A Cost-Effectiveness Analysis. J Altern Complement Med. 2018;24(6):564-569.

84. Wang Y, Xing Y, Chen L, et al. Fluconazole versus mould-active triazoles for primary antifungal prophylaxis in adult patients with acute lymphoblastic leukemia: clinical outcome and cost-effectiveness analysis. Int J Hematol. 2018;107(2):235-243.

85. Zhou J, Liu F, Jiang W, et al. Cost-Effectiveness of Jingshu Granules Compared to Placebo for the Treatment of Patients with Cervical Radiculopathy in China: A Decision-Tree Model Based on Randomized Controlled Trial. J Altern Complement Med. 2019;25(12):1183-1192.

86. Wang H, Yao G, Chen X, et al. Ipragliflozin as an add-on therapy in type 2 diabetes mellitus patients: An evidence-based pharmacoeconomics evaluation.

Diabetes Res Clin Pract. 2019;157:107867.

87. Zhang Y, Wang Y, Van Driel ML, et al. Network meta-analysis and pharmacoeconomic evaluation of antibiotics for the treatment of patients infected with complicated skin and soft structure infection and hospital-acquired or ventilator-associated penumonia. Antimicrob Resist Infect Control. 2019;8:72.

88. Zhou J, Wang Y, Jiang G. Oxycodone versus morphine for cancer pain titration: A systematic review and pharmacoeconomic evaluation. PLoS One. 2020;15(4):e0231763.

89. Liu L, Hong D, Ma K, et al. Cost-effectiveness analysis of cinacalcet for haemodialysis patients with moderate-to-severe secondary hyperparathyroidism in China: evaluation based on the EVOLVE trial. BMJ Open. 2020;10(8):e034123.

90. Chen Y, Lin L, Zhang H, et al. Pharmacoeconomic evaluation of dapagliflozin combined with conventional treatment of heart failure. Chinese Journal of Modern Applied Pharmacy. 2022;39(16):2151-2155.

91. Tian L, Xiong X, Guo Q, et al. Cost-Effectiveness of Tofacitinib for Patients with Moderate-to-Severe Rheumatoid Arthritis in China. Pharmacoeconomics. 2020;38(12):1345-1358.

92. Zeng F, Wang M, Zhang D. Cost-effectiveness analysis of 5% lidocaine-medicated plaster compared with pregabalin for the treatment of post-herpetic neuralgia in China. Ann Palliat Med. 2021;10(4):4493-4501.

93. Cui Z, Zhou W, Chang Q, et al. Cost-Effectiveness of Conbercept vs. Ranibizumab for Age-Related Macular Degeneration, Diabetic Macular Edema, and Pathological Myopia: Population-Based Cohort Study and Markov Model. Front Med (Lausanne). 2021;8:750132.

94. Sun KX, Cui B, Cao SS, et al. A meta-analysis and cost-minimization analysis of bivalirudin versus heparin in high-risk patients for percutaneous coronary intervention. Pharmacol Res Perspect. 2021;9(3):e00774.

95. Bao K, Li X, He X, et al. Pharmacoeconomic Evaluation of Erlotinib for the Treatment of Pancreatic Cancer. Clin Ther. 2021;43(6):1107-1115.

96. Jiang Y, Li Y, Wang LXW. Cost-effectiveness analysis of nivolumab plus standard chemotherapy versus chemotherapy alone for the first-line treatment of unresectable advanced or metastatic gastric cancer, gastroesophageal junction cancer, and esophageal adenocarcinoma. Int J Clin Pharm. 2022;44(2):499-506.

97. Jia S, Zhao M, Chen B, et al. Harmacoeconomic evaluation of rituximab (hanlikang) combined with CHOP chemotherapy regimen for diffuse large B-cell lymphoma. China Journal of Pharmaceutical Economics. 2019;14(10):20-27.

98. Mu S, Liu Y. Cost-effectiveness analysis of pantoprazole versus omeprazole in the treatment of gastric ulcer. Chinese Journal of Drug Evaluation. 2019;36(03):236-240.

99. Liu J, Wan C, Chen L, Xi X. Pharmacoeconomic evaluation of sodium tanshinone iia sulfonate in the treatment of angina pectoris of coronary heart disease. China Pharmacy. 2020;31(18):2240-2246.

100. Shi F, Ding L, Yao X, Wang C, Lei J, He W. The economic evaluation of antibiotics in the treatment of pulmonary infection with acinetobacter baumannii based on decision tree mode. Chinese General Practise. 2020;23(08):980-984.

101. Wang C, Han S. A short-term cost-effecttiveness analysis of nigroxine for the treatment of vascular dementia. China Journal of Pharmaceutical Economics. 2020;15(08):28-31.

102. Liu G, Kang S. Cost-utility analysis of atezolizumab combined with standard chemotherapy regimen in the first- line treatment of extensive-stage small-cell lung cancer. China Pharmacy. 2021;32(01):77-81.

103. Shi J, Qi R, Gao S, et al. Cost-utility analysis of durvalumab for consolidation therapy after chemoradiotherapy for stage III nonsmall cell lung cancer. China Pharmacy. 2022;33(15):1860-1864.

104. Wu Z, Zhao Q, Hai X. Cost-effectiveness analysis of the additional use of evolocumab in patients with atherosclerotic cardiovascular disease. Chinese Journal of Drug Application and Monitoring. 2021;18(02):79-83.

105. Yang G, Li Z, You W, et al. Comparison of efficacy, safety and pharmacoeconomics of morinidazole and ornidazole in treatment of anaerobic bacteria infection. Chinese Journal of New Drugs and Clinical Remedies. 2021;40(03):225-229.

106. Gong X. Economic evaluation of sacubitril/valsartan and enalapril in the treatment of heart failure——based on Markov model. China Journal of Pharmaceutical Economics. 2022;17(05):67-70.

107. Pang W, Zhu B, Li J, et al. Application and pharmacoeconomic study of drugs for delaying the progression of parkinson's disease in 30 tertiary hospitals from 2014 to 2019. Herald of Medicine. 2022,41(10):1513-1518.

108. Peng W, Gu H, Jiang C, et al. Comparison of tenofovir alafenamide and entecavir for hepatitis B virus-related acute-on-chronic liver failure. Journal of Central South University(Medical Science). 2022;47(02):194-201.

109. Pei B, Cao S, Zhang F, et al. Clinical observation and economic evaluation of cefoperazone - sulbactam combined with ornidazole in the treatment of acute suppurative appendicitis in children. Chinese Journal of Hospital Pharmacy. 2022;42(01):45-48.

110. Zhang C, Ran F, Gu L, et al. Cost-utility analysis of atezolizumab in the second-line treatment of metastatic non small cell lung cancer. Herald of Medicine. 2022;41(12):1854-1859.

111. Qi R, Du G, Liu X, et al. Cost-utility analysis of pembrolizumab combined with chemotherapy in the first-line treatment of advanced or metastatic esophageal carcinoma. China Pharmacy. 2022;33(12):1466-1473.

112. Li L, Zhao L, Zhong X, et al. Cost-effectiveness of camrelizumab added to chemotherapy in patients with advanced or metastatic esophageal squamous cell

carcinoma. Chinese Journal of New Drugs. 2022;31(24):2498-2503.

113. Gao S, Li X, Liu G. Pharmacoeconomic evaluation of pentoxifylline sustained release tablets in the treatment of cerebral infarction. China Journal of Pharmaceutical Economics. 2022;17(12):5-8.

114. Dai H, Wang W, Fan X, et al. Cost-effectiveness of camrelizumab plus chemotherapy vs. chemotherapy in the first-line treatment of non-squamous NSCLC: Evidence from China. Front Med (Lausanne). 2023;10:1122731.

115. Fei Z, Rui M, Wang Y, et al. Cost-effectiveness of anlotinib vs. pembrolizumab and nivolumab as third-line treatment in recurrent small cell lung cancer in China. Expert Rev Pharmacoecon Outcomes Res. 2023;23(1):79-87.

116. Gong H, Ong SC, Li F, et al. Cost-effectiveness analysis of sorafenib, lenvatinib, atezolizumab plus bevacizumab and sintilimab plus bevacizumab for the treatment of advanced hepatocellular carcinoma in China. Cost Eff Resour Alloc. 2023;21(1):20.

117. Hu S, Gu S, Qi C, et al. Cost-utility analysis of semaglutide for type 2 diabetes after its addition to the National Medical Insurance System in China. Diabetes Obes Metab. 2023;25(2):387-397.

118. Hu T, Liu Y, Lou Y. Sacubitril-valsartan versus enalapril for the treatment of acute decompensated heart failure in Chinese settings: A cost-effectiveness analysis. Front Pharmacol. 2023;14:925375.

119. Huang X, Liu Y, Lin S, et al. Adjuvant zoledronic acid therapy for postmenopausal women with early breast cancer in China: a cost-effectiveness analysis. Int J Qual Health Care. 2023;35(2):mzad016.

120. Huo G, Liu W, Kang S, et al. Toripalimab plus chemotherapy vs. chemotherapy in patients with advanced non-small-cell lung cancer: A cost-effectiveness analysis. Front Pharmacol. 2023;14:1131219.

121. Kong W, Yang X, Shu Y, et al. Cost-effectiveness analysis of ceftazidime-avibactam as definitive treatment for treatment of carbapenem-resistant Klebsiella pneumoniae bloodstream infection. Front Public Health. 2023;11:1118307.

122. Li N, Lei J, Zhang J, et al. Cost-effectiveness analysis of axicabtagene ciloleucel as a second-line treatment for diffuse large B-cell lymphoma in China and the United States. Ther Adv Hematol. 2023;14:20406207231168215.

123. Chen Z, Wang Y, Xing F. Pharmacoeconomic evaluation of Ningmitai Capsules in treatment of chronic prostatitis. Chinese Traditional and Herbal Drugs. 2023; 54(06):1906-1916.

124. Li W, Wan L. A trial-based cost-utility analysis of sugemalimab vs. placebo as consolidation therapy for unresectable stage III NSCLC in China. PLoS One. 2023;18(6):e0286595.

125. Li X, Cao Y. Cost-effectiveness of Arg16Gly in ADRB2 pharmacogenomic-guided treatment for pediatric asthma [published online ahead of print, 2023 Jun 5]. Expert Rev Pharmacoecon Outcomes Res. 2023;1-9.

126. Liang X, Chen X, Li H, et al. Tislelizumab plus chemotherapy is more cost-effective than chemotherapy alone as first-line therapy for advanced non-squamous non-small cell lung cancer. Front Public Health. 2023;11:1009920.

127. Lin Z, Zuo C, Jiang Y, et al. Cost-Effectiveness Analysis of Relmacabtagene Autoleucel for Relapsed or Refractory Large B-Cell Lymphoma in China. Value Health Reg Issues. 2023;37:41-48.

128. Liu L, Ruan Z, Ung COL, et al. Long-Term Cost-Effectiveness of Subcutaneous Once-Weekly Semaglutide Versus Polyethylene Glycol Loxenatide for Treatment of Type 2 Diabetes Mellitus in China. Diabetes Ther. 2023;14(1):93-107.

129. Liu S, Dou L, Li S. Cost-effectiveness analysis of PD-1 inhibitors combined with chemotherapy as first-line therapy for advanced esophageal squamous-cell

carcinoma in China. Front Pharmacol. 2023;14:1055727.

130. Liu S, Jiang N, Dou L, et al. Cost-effectiveness analysis of serplulimab plus chemotherapy in the first-line treatment for PD-L1-positive esophageal squamous cell carcinoma in China. Front Immunol. 2023;14:1172242.

131. Nie J, Wu H, Sun L, et al. Cost-effectiveness of fuzuloparib compared to routine surveillance, niraparib and olaparib for maintenance treatment of patients with germline BRCA1/2 mutation and platinum-sensitive recurrent ovarian carcinoma in China. Front Pharmacol. 2023;13:987337.

132. Qiu Y, Zha J, Ma A, et al. Cost-effectiveness analysis of niraparib maintenance therapy in Chinese patients with platinum-sensitive recurrent ovarian cancer. Gynecol Oncol. 2023;174:175-181.

133. Shang F, Zhang B, Kang S. Cost-effectiveness analysis of atezolizumab plus chemotherapy as first-line treatment for patients with advanced nonsquamous non-small-cell lung cancer in China. Expert Rev Pharmacoecon Outcomes Res. 2023;23(3):337-343.

134. Shao T, Zhao M, Liang L, et al. Serplulimab Plus Chemotherapy vs Chemotherapy for Treatment of US and Chinese Patients with Extensive-Stage Small-Cell Lung Cancer: A Cost-Effectiveness Analysis to Inform Drug Pricing. BioDrugs. 2023;37(3):421-432.

135. Shi X, Fu J, Li X, et al. Cost-effectiveness of ceftazidime/avibactam plus metronidazole versus meropenem as first-line empiric therapy for the treatment of complicated intra-abdominal infections: A study based on the in-vitro surveillance data in China. J Infect Public Health. 2023;16(3):361-367.

136. Shi Y, Qian D, Li Y, et al. Comparing the cost-effectiveness of sintilimab + pemetrexed plus platinum and pemetrexed plus platinum alone as a first-line therapy for Chinese patients with nonsquamous non-small cell lung cancer. Transl Cancer Res. 2023;12(4):928-938.

137. Shu Y, Ding Y, Li F, et al. Cost-effectiveness of nivolumab plus ipilimumab versus chemotherapy as first-line therapy in advanced non-small cell lung cancer. Int Immunopharmacol. 2023;114:109589.

138. Shu Y, Tang Y, Ding Y, et al. Cost-effectiveness of nivolumab versus sorafenib as first-line treatment for advanced hepatocellular carcinoma. Int Immunopharmacol. 2023;122:110543.
139. Wang L, Peng Y, Qin S, et al. First-line systemic treatment strategies for unresectable hepatocellular carcinoma: A cost-effectiveness analysis. PLoS One. 2023;18(4):e0279786.
140. Wu W, Ding S, Mingming Z, et al. Cost effectiveness analysis of CAR-T cell therapy for patients with relapsed/refractory multiple myeloma in China. J Med Econ. 2023;26(1):701-709.
141. Xu K, Wu H, Zhou C, et al. Cost-effectiveness of toripalimab plus chemotherapy for advanced esophageal squamous cell carcinoma. Int J Clin Pharm. 2023;45(3):641-649.
142. Ye Z, Zhou T, Zhang M, et al. Cost-effectiveness of endovascular thrombectomy with alteplase versus endovascular thrombectomy alone for acute ischemic stroke secondary to large vessel occlusion. CMAJ Open. 2023;11(3):E443-E450.
143. Lang Y, Zhu C, Tao W, et al. Pharmacoeconomic evaluation of clopidogrel versus aspirin for secondary prevention of ischemic stroke. China Pharmacy. 2023;34(07):837-843.
144. Qi R, Nie X, Liu X, et al. Pharmacoeconomic evaluation of serplulimab combined with chemotherapy regimens for the first-line treatment of extensive-stage small-cell lung cancer. China Pharmacy. 2023;34(11):1368-1373.
145. Yao W, Song X. Pharmacoeconomic evaluation of 2 kinds of inhalation schemes of budesonide for mild asthma. China Pharmacy. 2023;34(01):78-81.
146. Ye ZM, Xu Z, Li H, et al. Cost-effectiveness analysis of durvalumab plus chemotherapy as first-line treatment for biliary tract cancer. Front Public Health. 2023;11:1046424.

147. Zeng X, Jia Y, Chen H, et al. A real-world analysis of survival and cost-effectiveness of sintilimab plus bevacizumab biosimilar regimen in patients with advanced hepatocellular carcinoma. J Cancer Res Clin Oncol. 2023;149(11):9213-9219.

148. Zhang C, Liu Y, Tan J, et al. Cost-effectiveness evaluation based on two models of first-line atezolizumab monotherapy and chemotherapy for advanced non-small cell lung cancer with high-PDL1 expression. Front Oncol. 2023;13:1093469.

149. Zhang M, Xu K, Lin Y, et al. Cost-effectiveness analysis of toripalimab plus chemotherapy versus chemotherapy alone for advanced non-small cell lung cancer in China. Front Immunol. 2023;14:1169752.

150. Zhao Q, Xie R, Zhong W, et al. Cost-effectiveness analysis of adding durvalumab to chemotherapy as first-line treatment for advanced biliary tract cancer based on the TOPAZ-1 trial. Cost Eff Resour Alloc. 2023;21(1):19.

151. Zhou K, Shu P, Zheng H, et al. Cost-effectiveness analysis of toripalimab plus chemotherapy as the first-line treatment in patients with advanced non-small cell lung cancer (NSCLC) without EGFR or ALK driver mutations from the Chinese perspective. Front Pharmacol. 2023;14:1133085.

152. Zhu W, Zheng M, Xia P, et al. Cost-effectiveness of palbociclib plus fulvestrant as second-line therapy of women with HR+/HER2- advanced breast cancer - A Chinese healthcare system perspective. Front Oncol. 2023;13:1068463.

153. Zhu Y, Liu K, Qin Q, et al. Serplulimab plus chemotherapy as first-line treatment for extensive-stage small-cell lung cancer: A cost-effectiveness analysis. Front Immunol. 2023;13:1044678.

154. Wang C, Li W, Cui X, et al. Pharmacoeconomic evalution of lienal polypeptide combined with chemotherapy for advanced non-small lung cancer. Chinese Journal of Hospital Pharmacy. 2023;43(05):525-529.
