# Supplementary material for: A Systematic Review and Quality Assessment of Pharmacoeconomic Publications for China Compared to Internationally: Is the Quality of Evidence-Base Sufficient for Health Technology Assessment?
Source: Int J Health Policy Manag. 2025 Apr 28;14:8656. doi: 10.34172/ijhpm.8656 (PMC12257205; doi:10.34172/ijhpm.8656)
Supplement: Supplementary file 4 — describes the CHEERS 2013/2022 checklist scores and AMSTAR 2 scores for the articles included in the umbrella review. [file ijhpm-14-8656-s004.pdf]

Article title: A Systematic Review and Quality Assessment of Pharmacoeconomic Publications for China Compared to Internationally: Is the Quality of Evidence-Base Sufficient for Health Technology Assessment?

Journal name: International Journal of Health Policy and Management

Authors' information: Zhixin Fan[1,2,3]¶, Xu Si[1,2,3]¶, Zhongxiang Wang[4], Liwei Zhang[1,2,3], Junyang Liu[1,2,3], Qing He[1,2,3], Matthew Franklin[5], Qiang Sun[1,2,3,6]*, Jia Yin[1,2,3]*

[1]Department of Social Medicine and Health Management, School of Public Health, Cheeloo College of Medicine, Shandong University, Jinan, China.

[2]NHC Key Lab of Health Economics and Policy Research, Shandong University, Jinan, China.

[3]Center for Health Management and Policy Research, Shandong University, Shandong Provincial Key New Think Tank, Jinan, China.

[4]Zhucheng Shiqiaozi Health Hospital, Zhucheng, China.

[5]Health Economics and Decision Science (HEDS), School of Health and Related Research (ScHARR), University of Sheffield, Sheffield, UK.

[6]China National Health Development Research Center, Beijing, China.

***Correspondence to:** Qiang Sun, qiangs@sdu.edu.cn & Jia Yin, yinjia@sdu.edu.cn

¶ Both authors contributed equally to this paper.

Citation: Fan Z, Si X, Wang Z, et al. A systematic review and quality assessment of pharmacoeconomic publications for China compared to internationally: Is the quality of evidence-base sufficient for health technology assessment? Int J Health Policy Manag. 2025;14:8656. doi:10.34172/ijhpm.8656

Supplementary file 4

1. CHEERS 2013 checklist scores extracted of studies included the umbrella review

Table 1. CHEERS 2013 checklist scores extracted of studies included the umbrella review.

Article		Asma Rashki Kemmak,elt.	Silvia Marquez-Megias,elt.	Hui-Yao Huang, elt.	Iara Carolina Barbosa Henrique, elt.	Dina Hamdi Abushanab, elt.	Ahmad Al Kadour, elt.
Number of included articles		6	13	17	17	7	21
Item 1	Yes	6	10	14	8	7	18
	Partial Yes	0	0	1	0	0	3
	No	0	3	2	9	0	0
	Not applicable	0	0	0	0	0	0
Item 2	Yes	6	9	15	7	7	14
	Partial Yes	0	0	0	0	0	7
	No	0	4	2	10	0	0
	Not applicable	0	0	0	0	0	0
Item 3	Yes	6	13	17	11	7	17
	Partial Yes	0	0	0	0	0	4
	No	0	0	0	6	0	0
	Not applicable	0	0	0	0	0	0
Item 4	Yes	6	10	15	3	6	20
	Partial Yes	0	0	0	0	1	1
	No	0	3	2	14	0	0
	Not applicable	0	0	0	0	0	0
Item 5	Yes	6	8	17	17	3	19
	Partial Yes	0	0	0	0	4	2
	No	0	5	0	0	0	0
	Not applicable	0	0	0	0	0	0
Item 6	Yes	6	6	15	13	7	18
	Partial Yes	0	0	0	0	0	2
	No	0	7	2	4	0	0
	Not applicable	0	0	0	0	0	1
Item 7	Yes	6	11	16	13	7	21
	Partial Yes	0	0	0	0	0	0
	No	0	2	1	4	0	0
	Not applicable	0	0	0	0	0	0

Item 8	Yes	6	3	16	8	2	12
	Partial Yes	0	0	0	0	0	7
	No	0	10	1	9	0	0
	Not applicable	0	0	0	0	5	2
Item 9	Yes	5	4	14	5	0	10
	Partial Yes	0	0	0	0	0	6
	No	1	9	3	12	0	0
	Not applicable	0	0	0	0	0	5
Item 10	Yes	6	9	2	0	6	3
	Partial Yes	0	0	0	0	0	18
	No	0	4	0	17	0	0
	Not applicable	0	0	15	0	1	0
Item 11	Yes	6	4	2	17	7	10
	Partial Yes	0	0	0	0	0	10
	No	0	9	0	0	0	0
	Not applicable	0	0	15	0	0	1
Item 12	Yes	6	9	0	13	0	0
	Partial Yes	0	0	0	0	0	0
	No	0	4	1	0	0	0
	Not applicable	0	0	16	4	0	0
Item 13	Yes	6	7	16	15	2	17
	Partial Yes	0	0	0	0	5	4
	No	0	6	1	2	0	0
	Not applicable	0	0	0	0	0	0
Item 14	Yes	5	6	14	12	2	13
	Partial Yes	0	0	0	0	4	8
	No	1	7	3	5	0	0
	Not applicable	0	0	0	0	1	0
Item 15	Yes	6	8	0	9	4	7
	Partial Yes	0	0	0	0	0	10
	No	0	5	0	8	0	0
	Not applicable	0	0	17	0	3	4
Item 16	Yes	3	8	0	17	3	11
	Partial Yes	3	0	0	0	0	6

	No	0	5	0	0	0	0
	Not applicable	0	0	17	0	4	4
Item 17	Yes	6	9	17	17	4	10
	Partial Yes	0	0	0	0	0	9
	No	0	4	0	0	0	0
	Not applicable	0	0	0	0	3	2
Item 18	Yes	6	9	17	2	7	17
	Partial Yes	0	0	0	0	0	4
	No	0	4	0	15	0	0
	Not applicable	0	0	0	0	0	0
Item 19	Yes	6	8	0	10	1	16
	Partial Yes	0	0	0	0	0	2
	No	0	5	0	7	0	0
	Not applicable	0	0	17	0	6	3
Item 20	Yes	6	4	10	15	2	16
	Partial Yes	0	0	0	0	0	2
	No	0	9	7	2	0	0
	Not applicable	0	0	0	0	5	3
Item 21	Yes	1	3	4	0	2	0
	Partial Yes	1	0	0	0	0	2
	No	4	10	5	0	0	0
	Not applicable	0	0	8	17	5	19
Item 22	Yes	3	12	15	12	0	8
	Partial Yes	1	0	2	0	7	13
	No	2	1	0	5	0	0
	Not applicable	0	0	0	0	0	0
Item 23	Yes	5	9	13	7	0	7
	Partial Yes	0	0	0	0	0	9
	No	1	4	4	10	0	0
	Not applicable	0	0	0	0	7	5
Item 24	Yes	4	11	15	16	0	9
	Partial Yes	0	0	0	0	0	2
	No	2	2	2	1	0	0
	Not applicable	0	0	0	0	7	10

2. CHEERS 2022 checklist scores extracted of studies included the umbrella review

Table 2. CHEERS 2022 checklist scores extracted of studies included the umbrella review.

Article		Parand Rostamian, elt.(N=10)	Guangyi Yu, elt.(N=10)			Parand Rostamian, elt. .(N=10)	Guangyi Yu, elt. .(N=10)			Parand Rostamian, elt. .(N=10)	Guangyi Yu, elt. .(N=10)			Parand Rostamian, elt. .(N=10)	Guangyi Yu, elt. .(N=10)
Item 1	Yes	10	10	Item 8	Yes	9	10	Item 15	Yes	9	9	Item 22	Yes	10	10
	Partial Yes	0	0		Partial Yes	1	0		Partial Yes	1	1		Partial Yes	0	0
	No	0	0		No	0	0		No	0	0		No	0	0
	Not applicable	0	0		Not applicable	0	0		Not applicable	0	0		Not applicable	0	0
Item 2	Yes	10	10	Item 9	Yes	10	10	Item 16	Yes	9	10	Item 23	Yes	10	10
	Partial Yes	0	0		Partial Yes	0	0		Partial Yes	0	0		Partial Yes	0	0
	No	0	0		No	0	0		No	1	0		No	0	0
	Not applicable	0	0		Not applicable	0	0		Not applicable	0	0		Not applicable	0	0
Item 3	Yes	10	10	Item 10	Yes	9	10	Item 17	Yes	9	10	Item 24	Yes	2	10
	Partial Yes	0	0		Partial Yes	1	0		Partial Yes	1	0		Partial Yes	5	0
	No	0	0		No	0	0		No	0	0		No	3	0
	Not applicable	0	0		Not applicable	0	0		Not applicable	0	0		Not applicable	0	0
Item 4	Yes	10	10	Item 11	Yes	10	10	Item 18	Yes	6	8	Item 25	Yes	1	0
	Partial Yes	0	0		Partial Yes	0	0		Partial Yes	2	1		Partial Yes	5	0
	No	0	0		No	0	0		No	2	1		No	4	10
	Not applicable	0	0		Not applicable	0	0		Not applicable	0	0		Not applicable	0	0
Item 5	Yes	9	10	Item 12	Yes	7	10	Item 19	Yes	7	0	Item 26	Yes	10	0
	Partial Yes	1	0		Partial Yes	3	0		Partial Yes	2	0		Partial Yes	0	10
	No	0	0		No	0	0		No	1	10		No	0	0

	Not applicable	0	0		Not applicable	0	0		Not applicable	0	0		Not applicable	0	0
Item 6	Yes	0	0	Item 13	Yes	9	10	Item 20	Yes	9	10	Item 27	Yes	9	0
	Partial Yes	5	0		Partial Yes	0	0		Partial Yes	0	0		Partial Yes	1	0
	No	1	0		No	1	0		No	1	0		No	0	3
	Not applicable	0	0		Not applicable	0	0		Not applicable	0	0		Not applicable	0	0
Item 7	Yes	9	10	Item 14	Yes	9	10	Item 21	Yes	8	0	Item 28	Yes	8	7
	Partial Yes	1	0		Partial Yes	0	0		Partial Yes	1	0		Partial Yes	0	0
	No	0	0		No	1	0		No	1	10		No	2	3
	Not applicable	0	0		Not applicable	0	0		Not applicable	0	0		Not applicable	0	0

3. AMSTAR 2 scores for articles included in the umbrella review

Table 5. AMSTAR 2 scores for articles included in the umbrella review

**Articles**	Asma Rashki Kemmak,elt.	Silvia Marquez-Megias,elt.	Hui-Yao Huang, elt.	Iara Carolina Barbosa Henrique, elt.	Dina Hamdi Abushanab, elt.	Ahmad Al Kadour, elt.	Parand Rostamian, elt.	Guangyi Yu, elt.
Time	2022	2022	2020	2020	2019	2018	2023	2023
Number of included articles	6	13	17	17	8	21	10	10
Item 1. Did the research questions and inclusion criteria for the review include the components of PICO?	Yes	Yes	Yes	Yes	Yes	Yes	Yes	Yes
Item 2. Did the report of the review contain an explicit statement that the review methods were established prior to the conduct of the review and did the report justify any significant deviations from the protocol?	No	No	No	No	No	No	No	No
Item 3.Did the review authors explain their selection of the study designs for inclusion in the review?	Yes	Yes	Yes	Yes	Yes	Yes	Yes	Yes
Item 4. Did the review authors use a comprehensive literature search	Yes	Yes	Yes	Yes	Yes	Yes	Partial Yes	Yes

strategy?								
Item 5. Did the review authors perform study selection in duplicate?	Yes	Yes	Yes	Yes	Yes	Yes	Yes	Yes
Item 6. Did the review authors perform data extraction in duplicate?	Yes	Yes	Yes	Yes	Yes	Yes	Yes	Yes
Item 7. Did the review authors provide a list of excluded studies and justify the exclusions?	No	Yes	No	No	No	Yes	No	Partial Yes
Item 8. Did the review authors describe the included studies in adequate detail?	Yes	Yes	Yes	Yes	Yes	Yes	Yes	Yes
Item 9. Did the review authors use a satisfactory technique for assessing the risk of bias (RoB) in individual studies that were included in the review?	Yes	Yes	Partial Yes	Partial Yes	Yes	Yes	Partial Yes	Partial Yes
Item 10.Did the review authors report on the sources of funding for the studies included in the review?	Yes	Yes	Yes	Yes	Yes	Yes	Yes	Yes
Item 11.If meta-analysis was performed, did the review authors use appropriate methods for statistical combination of results?	No	No	No	No	No	No	No	No
Item 12.If meta-analysis was performed, did the review authors assess the potential impact of RoB in individual studies on the results of the meta-analysis or other evidence synthesis?	No	No	No	No	No	No	No	No
Item 13.Did the review authors account for RoB in primary studies when interpreting/discussing the results of the review?	No	Yes	Yes	Yes	Yes	Yes	No	No
Item 14.Did the review authors provide a satisfactory explanation for, and discussion of, any heterogeneity observed in the results of the review?	No	Partial Yes	Yes	Partial Yes	Partial Yes	Partial Yes	Partial Yes	Partial Yes
Item 15. If they performed quantitative synthesis did the review authors carry out an adequate investigation of publication bias (small study bias) and discuss its likely impact on the results of the review?	No	No	No	No	No	No	No	No
Item 16.Did the review authors report any potential sources of conflict	Yes	Yes	Yes	Yes	Yes	Yes	Yes	Yes

of interest, including any funding they received for conducting the review?								
