# Supplementary material for: A Systematic Review and Quality Assessment of Pharmacoeconomic Publications for China Compared to Internationally: Is the Quality of Evidence-Base Sufficient for Health Technology Assessment?
Source: Int J Health Policy Manag. 2025 Apr 28;14:8656. doi: 10.34172/ijhpm.8656 (PMC12257205; doi:10.34172/ijhpm.8656)
Supplement: Supplementary file 5 — describes the lists of studies included in the umbrella review and the basic information of them. [file ijhpm-14-8656-s005.pdf]

Article title: A Systematic Review and Quality Assessment of Pharmacoeconomic Publications for China Compared to Internationally: Is the Quality of Evidence-Base Sufficient for Health Technology Assessment?

Journal name: International Journal of Health Policy and Management

Authors' information: Zhixin Fan[1,2,3]¶, Xu Si[1,2,3]¶, Zhongxiang Wang[4], Liwei Zhang[1,2,3], Junyang Liu[1,2,3], Qing He[1,2,3], Matthew Franklin[5], Qiang Sun[1,2,3,6]*, Jia Yin[1,2,3]*

[1]Department of Social Medicine and Health Management, School of Public Health, Cheeloo College of Medicine, Shandong University, Jinan, China.

[2]NHC Key Lab of Health Economics and Policy Research, Shandong University, Jinan, China.

[3]Center for Health Management and Policy Research, Shandong University, Shandong Provincial Key New Think Tank, Jinan, China.

[4]Zhucheng Shiqiaozi Health Hospital, Zhucheng, China.

[5]Health Economics and Decision Science (HEDS), School of Health and Related Research (ScHARR), University of Sheffield, Sheffield, UK.

[6]China National Health Development Research Center, Beijing, China.

***Correspondence to:** Qiang Sun, qiangs@sdu.edu.cn & Jia Yin, yinjia@sdu.edu.cn

¶ Both authors contributed equally to this paper.

Citation: Fan Z, Si X, Wang Z, et al. A systematic review and quality assessment of pharmacoeconomic publications for China compared to internationally: Is the quality of evidence-base sufficient for health technology assessment? Int J Health Policy Manag. 2025;14:8656. doi:10.34172/ijhpm.8656

Supplementary file 5

1. Lists of studies included in umbrella review.

1. Rashki Kemmak A, Dolatshahi Z, Mezginejad F, et al. Economic evaluation of ivabradine in treatment of patients with heart failure: a systematic review. Expert Rev Pharmacoecon Outcomes Res. 2022;22(1):37-44.

2. Marquez-Megias S, Nalda-Molina R, Sanz-Valero J, et al. Cost-Effectiveness of Therapeutic Drug Monitoring of Anti-TNF Therapy in Inflammatory Bowel Disease: A Systematic Review. Pharmaceutics. 2022;14(5):1009. Published 2022 May 7.

3. Huang HY, Liu CC, Yu Y, et al. Pharmacoeconomic Evaluation of Cancer Biosimilars Worldwide: A Systematic Review. Front Pharmacol. 2020;11:572569. Published 2020 Nov 12.

4. Henrique ICB, de Mendonça Lima T, de Melo DO, et al. Economic evaluations on the use of aripiprazole for patients with schizophrenia: A systematic review. J Clin Pharm Ther. 2020;45(1):1-15.

5. Abushanab DH, Alsoukhni OA, Al-Badriyeh D. Evaluations of Morphine and Fentanyl for Mechanically Ventilated Patients With Respiratory Disorders in Intensive Care: A Systematic Review of Methodological Trends and Reporting Quality. Value Health Reg Issues. 2019;19:7-25.

6. Al Kadour A, Marridi WA, Al-Badriyeh D. Pharmacoeconomics Evaluations of Oral Anticancer Agents: Systematic Review of Characteristics, Methodological Trends, and Reporting Quality. Value Health Reg Issues. 2018;16:46-60.

7. Mohammadnezhad G, Noqani H, Rostamian P, et al. Lenvatinib in the treatment of unresectable hepatocellular carcinoma: a systematic review of economic evaluations. Eur J Clin Pharmacol. 2023;79(7):885-895.

8. Yu G, Tong S, Liu J, et al. A systematic review of cost-effectiveness analyses of sequential treatment for osteoporosis. Osteoporos Int. 2023;34(4):641-658.

2. Basic information of studies included umbrella review

Study	Year of publication	Time range	Country	Distribution of diseases	Perspective	Types of costs	Discount rate	Model	Outcomes	Incremental analysis (Yes/No)	Sensitivity analysis
Rashki et al.	2022	2014-2020	Iran, Thailand, Australia, US, UK, and Greece	Chronic heart failure	Health-care system(1/3), Health-care payers(1/3), Third-party payer(1/3)	Direct medical cost	3%–7.2%	Markov	QALYs	Yes	One-way sensitivity analyses & PSA
Marquez-Megias et al.	2022	From inception to 2021	US, UK, France, Australia, Italy, The Netherlands, Belgium, Denmark	Inflammatory bowel diseases	—	Direct medical cost	—	Markov model, stochastic simulation model, discrete event model	QALYs NA	Yes	—
Huang et al.	2020	From inception to 2019	European countries (13 studies) and the United States (4 studies)	Oncology biosimilars	Payers	Direct medical cost	—	—	CMA	No	One-way sensitivity analyses
Henrique et al.	2020	From inception to March 2018	Scotland and Wales, France, Singapore,	Schizophrenia	NHS and social services, Ministry of Health,	Direct medical costs	3%-5%	Markov, Monte Carlo microsimulation, Decision	QALYs, Life-years, relapse-free days, Relapse avoided	Yes	Deterministic and probabilistic

			Finland, Canada, US, Spain, Sweden, Greece, Slovenia		Ministry of Health and societal， Payers			tree			
Abushanab et al.	2019	From inception to Sept 2017	US, Spain, Denmark, Canada, Brazil, Australia, Argentina, New Zealand, Malaysia, China, Ireland	Respiratory disorders	hospital perspective	Direct medical costs extra costs of additional care	_	Markov, Decision tree	Treatment success based on the desired level of sedation using RSS, Apnea and respiratory effort, Weaning time and cost, Duration of MV, ICU, and hospital stay, et al.	Yes	_
Al Kadour et al.	2018	From inception to 2017	US, UK, Japan, China, Italy, Sweden, Finland, Canada, France, Spain	Oral Anticancer Agents	Payers Societal	Direct medical costs	_	Non-Markovian decision-analytic model, Markov	Total gain in life-years, progression-free life-years, and quality-adjusted life-years (QALYs)	Yes	deterministic and/or probabilistic one-way sensitivity analyses
Mohammadnezhad et al.	2023	From inception to July 2022	China, Japan, Australia, US, Canada	Hepatocellular carcinoma	hospital perspective payers	Direct medical costs	1.5-5%	Markov, Decision tree	QALYs and progression-free survival	Yes	One-way sensitivity analysis, PSA
Yu G et al.	2023	From inception to June 2022	US, Japan, China, Sweden	Osteoporosis	hospital perspective Payers Ministry of Health and societal，	Direct and indirect cost	1.5-5%	Markov, discrete event model	QALYs	Yes	One-way sensitivity analysis, PSA
